# Salivary Tick Cystatin OmC2 Targets Lysosomal Cathepsins S and C in Human Dendritic Cells

**DOI:** 10.3389/fcimb.2017.00288

**Published:** 2017-06-30

**Authors:** Tina Zavašnik-Bergant, Robert Vidmar, Andreja Sekirnik, Marko Fonović, Jiří Salát, Lenka Grunclová, Petr Kopáček, Boris Turk

**Affiliations:** ^1^Department of Biochemistry, Molecular and Structural Biology, Jožef Stefan InstituteLjubljana, Slovenia; ^2^Institute of Parasitology, Biology Centre of the Czech Academy of SciencesČeské Budějovice, Czechia; ^3^Centre of Excellence for Integrated Approaches in Chemistry and Biology of ProteinsLjubljana, Slovenia; ^4^Faculty of Chemistry and Chemical Technology, University of LjubljanaLjubljana, Slovenia

**Keywords:** cystatin OmC2, tick saliva, cathepsin S, cathepsin C, lysosomal proteases, DPP1, dipeptidyl peptidase 1, dendritic cells

## Abstract

To ensure successful feeding tick saliva contains a number of inhibitory proteins that interfere with the host immune response and help to create a permissive environment for pathogen transmission. Among the potential targets of the salivary cystatins are two host cysteine proteases, cathepsin S, which is essential for antigen- and invariant chain-processing, and cathepsin C (dipeptidyl peptidase 1, DPP1), which plays a critical role in processing and activation of the granule serine proteases. Here, the effect of salivary cystatin OmC2 from *Ornithodoros moubata* was studied using differentiated MUTZ-3 cells as a model of immature dendritic cells of the host skin. Following internalization, cystatin OmC2 was initially found to inhibit the activity of several cysteine cathepsins, as indicated by the decreased rates of degradation of fluorogenic peptide substrates. To identify targets, affinity chromatography was used to isolate His-tagged cystatin OmC2 together with the bound proteins from MUTZ-3 cells. Cathepsins S and C were identified in these complexes by mass spectrometry and confirmed by immunoblotting. Furthermore, reduced increase in the surface expression of MHC II and CD86, which are associated with the maturation of dendritic cells, was observed. In contrast, human inhibitor cystatin C, which is normally expressed and secreted by dendritic cells, did not affect the expression of CD86. It is proposed that internalization of salivary cystatin OmC2 by the host dendritic cells targets cathepsins S and C, thereby affecting their maturation.

## Introduction

As external parasites, ticks have adapted to their hosts to successfully feed while warding off the host immune system. The saliva of ticks contains multiple proteins with anti-haemostatic, anti-inflammatory and immunomodulatory properties (Díaz-Martin et al., 2013). Tick saliva influences the production and secretion of numerous cytokines, often simultaneously, from various types of immune cells (Kotál et al., 2015). The tick salivary proteins not only inhibit the components of the host immune system (Francischetti et al., 2009) but also have an impact on the transmission of vector-borne pathogens. Specifically, many species of *Borrelia* causing relapsing fever in infected humans, are transmitted by the soft ticks of *Ornithodoros genus* (Parola and Raoult, 2001). Multiple species of hard and soft ticks inhibit the production of various cytokines such as TNF-α, IL-6, IL-12, IL-17, and IFN-γ (Sá-Nunes et al., 2009; Fialová et al., 2010; Wu et al., 2010). Further, salivary molecules from *O. moubata* can modulate the host defense system by acting as platelet-aggregation inhibitors [e.g., moubatin (Waxman and Connolly, 1993) and disaggregin (Karczewski et al., 1994)], as complement inhibitors [e.g., OmCI (Nunn et al., 2005)], or by interfering with coagulation and fibrinolysis [e.g., ornithodorin (van de Locht et al., 1996) and tick anticoagulant peptide TAP (Waxman et al., 1990)].

Modulation of the proteolytic activity of targeted host cysteine proteases in antigen-presenting cells (APC) in the host skin may represent an additional mechanism utilized by ticks to modulate the immune response of their host. Cystatin OmC2 is naturally present in the saliva, salivary glands and gut of the soft tick *O. moubata* (Grunclová et al., 2006). Recombinant cystatin OmC2 was screened against a panel of recombinant lysosomal proteases. It was shown to be a broad-specificity inhibitor of mammalian cysteine cathepsins but not of mammalian legumain (asparaginyl endopeptidase), another cysteine protease potentially involved in the processing of antigens in APC. The crystal structure of cystatin OmC2 was determined and used to describe the structure-inhibitory activity relationship (Salát et al., 2010). The biological impact of cystatin OmC2 was demonstrated in mice both using *in vitro* and vaccination experiments. Cystatin OmC2 decreased the levels of TNF-α and IL-12 produced by LPS-activated dendritic cells (DC), as well as it was able to reduce the DC-mediated proliferation of naive CD4^+^ T cells. In addition, the vaccination of mice with recombinant cystatin OmC2 decreased the success of tick feeding. Ticks that fed on the mice with the highest level of anti-cystatin OmC2 antibodies had the highest mortality (Salát et al., 2010).

This study focused on two lysosomal cathepsins, S and C, which are both members of the papain-like superfamily of cysteine proteases (Turk et al., 2012; Rawlings et al., 2016). The first target, cathepsin S, is involved in the crucial step in the processing of major histocompatibility complex class II (MHC II)-associated chaperone invariant chain (Roche and Furuta, 2015; Lindner, 2017), as well as being involved in the lysosome-mediated response to microbial DNA *via* the TLR9 pathway (Matsumoto et al., 2008). The second target, cathepsin C, is an exoprotease, the function of which in human DC is not yet fully known. The structure of cathepsin C tetramer reveals the tetrahedral arrangement of its active sites. Each monomer consists of three domains: two domains consist of a papain-like structure that contains the catalytic site and an additional exclusion domain that provides features that endow cathepsin C with a dipeptidyl aminopeptidase activity (Dahl et al., 2001; Turk et al., 2001). The general function of dipeptidase cathepsin C is likely to be the degradation of the protein cargo in lysosomes as well as the processing of a diverse set of precursor proteins, which includes proteases. In neutrophils, cathepsin C is essential for the activation of the granule-associated serine proteases. These proteinases require the activity of cathepsin C to remove their propeptides and thus become active. Serine proteases, which are stored in granules in their active forms until they are released after neutrophil exposure to activating stimuli, exhibit broad biological effects including intracellular microbial killing and modulation of the recruitment of inflammatory cells (Pham, 2006). Cathepsin C is involved in the activation of elastase and cathepsin G in neutrophils and chymase and tryptase in mast cells (Pham and Ley, 1999; Pham, 2006; Guarino et al., 2017). Peripheral blood neutrophils upregulate their granzyme B expression and secretion when they are infected with *Mycobacterium tuberculosis* (Mattila et al., 2015). In addition, in natural killer (NK) cells (Maher et al., 2014) and T lymphocytes, cathepsin C is involved in the activation of progranzymes A and B into the proteolytically active enzymes. In patients with neutrophilic lung inflammation, mature cathepsin C is found in large amounts in sputa. It is secreted by activated neutrophils as confirmed through lipopolysaccharide (LPS) administration in a non-human primate model (Hamon et al., 2016). In cathepsin C-deficient mice, the function of the cytotoxic T cells is impaired (Pham and Ley, 1999) whereas in humans, defects in the cathepsin C gene are associated with Papillon-Lefevre disease, Haim-Munk syndrome (Sulák et al., 2016) and aggressive periodontitis (Nagy et al., 2014).

Pathogen invasion together with the proinflammatory signals drives the maturation of skin immature DC which then upregulate the expression of their costimulatory molecules and proinflammmatory cytokines. Distinct subsets of functionally specialized Langerhans cells (LC) and dermal DC have been distinguished among DC in epidermis and dermis (reviewed in Clausen and Stoitzner, 2015). In healthy mouse skin the DC network includes Langerin^+^CD11b^+^ LC, Langerin^+^CD11b^neg^CD103^+^ dermal DC, Langerin^+^CD11b^neg^CD103^neg^ dermal DC, Langerin^neg^CD11b^+^ dermal DC, and Langerin^neg^CD11b^low^XCR1^neg^ dermal DC. Human skin DC counterparts include: Langerin^+^CD1a^high^ LC, CD141^high^XCR1^+^ dermal DC and CD1c^+^CD1a^+^ dermal DC. The human acute myeloid leukemia cell line MUTZ-3 can be differentiated *in vitro* to dermal DC in the presence of GM-CSF, TNF-α and IL-4, and to LC in the presence of GM-CSF, TNF-α and TGF-β (Santegoets et al., 2008). MUTZ-3-derived DC, which have the great advantage of not being dependent on the donor material, express adequate immune-related transcripts (Larsson et al., 2006; Lundberg et al., 2013), supporting their suitability in immune applications (Santegoets et al., 2008). MUTZ-3-derived immature DC differentiated with IL-4 and cultured in the presence of LPS efficiently stimulated the proliferation of CD4^+^CD45RA^+^ T cells (Larsson et al., 2006) but failed to stimulate the proliferation of allogenic NK cells (Kim et al., 2006). In addition, MUTZ-3 cells that are differentiated with TGF-β to MUTZ-3-derived LC (MUTZ-LC) are often applied in the area of risk assessment of chemicals, in allergen and irritant exposure studies etc. (Kosten et al., 2015).

We have further evaluated the possibility of cystatin OmC2 to mimic the function of its host cystatin type 2 homologs, if successfully internalized to the host immune cells. Differentiated MUTZ-3 cells were applied as an *in vitro* cell model of human immature DC and tick cystatin OmC2 was evaluated whether it could interfere with the host proteolytic capacity mediated by the lysosomal cysteine cathepsins. We show that following its internalization *via* the endocytic pathway, cystatin OmC2 affected the activity of lysosomal cathepsins S and C, two key cysteine proteases of DC proteolytic machinery.

## Materials and methods

### Tick cystatin OmC2

The baculovirus expression system was used to prepare the salivary cystatin OmC2 as a recombinant protein with an oligo His-tag added to its C-terminus in insect Sf9 cells (Salát et al., 2010). Salivary tick cystatin OmC2 was also produced in *E. coli* (Grunclová et al., 2006), and this protein was used in the experiments, which are included in Supplementary Figures 1A,B. In addition, the recombinant tick inhibitor OmC2 from insect cells was fluorescently labeled with Alexa Fluor 488 dye (Alexa Fluor 488 Microscale Protein Labeling Kit, Life Technologies-Molecular Probes). The unbound Alexa Fluor 488 dye was removed from reaction mixture using size-exclusion chromatography (as recommended by the producer), additional dialysis and filtration by using Microcon-3 kDa (Millipore). The fluorescence was measured with Tecan microplate reader after each purification step. The labeled cystatin OmC2 was excited at 488 nm, and the fluorescence was followed using a confocal laser scanning microscope Leica SP5 X with a white light laser (Leica MicroSystems) or a Typhoon Variable Mode Imager (GE Healthcare). Prior to the application of the recombinant cystatin OmC2 in the cell studies, its inhibitory activity was determined by active site titration of 10 nM papain (Sigma-Aldrich) in 0.1 M phosphate buffer (pH 6.0). The active concentration of the papain was determined by titration with a synthetic inhibitor E-64 (Sigma-Aldrich). For these assays, 10 μM of the fluorogenic substrate Z-Phe-Arg-AMC (Bachem) was applied, and the fluorescence signal was measured using a Tecan Safire microplate reader. Fluorescently labeled cystatin OmC2 was applied when its internalization was followed by confocal microscopy in viable cells. When cells were assayed for activity measurements or pull-down experiment and mass spectrometry analysis, non-labeled cystatin OmC2 was applied. In addition, the complex formation between cystatin C and cathepsin S was evaluated *in vitro* using IEF. Human recombinant cystatin C was prepared in-house according to published procedures (Cimerman et al., 1999). Software from CBS (CBS Prediction Servers/Post-translational modifications of proteins/NetNGlyc, www.cbs.dtu.dk) was used for the prediction of possible O-glycosylation sites in cystatin OmC2.

### Cathepsins

Human recombinant cathepsin S and cathepsin L were expressed in the methylotrophic yeast *Pichia pastoris* and were isolated as previously reported (Mihelič et al., 2008). Human recombinant procathepsin C was expressed in insect Sf9 cells using the baculovirus expression system according to the procedure described elsewhere (Dahl et al., 2001) and was purified by using the modified procedure described by Poreba et al. (2014). Briefly, the cell-free culture medium was collected after 3 days, concentrated by ultrafiltration and dialyzed against 50 mM HEPES with 0.5 M NaCl, pH 7.5. Isolation of procathepsin C (containing an oligo His-tag added to the C-terminus) was performed using immobilized-metal (Ni) affinity chromatography (IMAC) from Roche (cOmplete His-Tag Purification Resin). A 300 mM imidazole solution was used to elute the procathepsin C. The protein was preserved in 30 mM TRIS buffer with 0.4 M NaCl (pH 7.5), or alternatively, 50% glycerol was added to avoid freezing at −20°C. The procathepsin C expression was confirmed by mass spectrometry using an UltrafleXtreme™ III MALDI-TOF/TOF instrument (Bruker). The procathepsin C was then proteolytically processed and activated under acidic condition by the proteases present in the insect cells or by recombinant human cathepsin L, the latter being added at a 1:20 molar ratio. The aminopeptidase activity of the purified cathepsin C was measured as described (Poreba et al., 2014).

### Isoelectric focusing (IEF)

Cathepsins S, C, or L were first activated with 5 mM DTT in 0.1 M phosphate buffer (pH 6.0; cathepsins S and C) or 0.1 M acetate buffer (pH 5.6; cathepsin L) for 5 min. The active cathepsins were next incubated with tick cystatin OmC2 or with human cystatin C at a 1:1 molar ratio for 10 additional min at 37°C. IEF was then performed using the XCell SureLock Mini-Cell system (Invitrogen-Life Technologies). The proteins were separated on vertical pre-cast IEF gels (pH 3–7 and pH 3–10) from Invitrogen. IEF Markers 3–10 (SERVA Liquid Mix) were applied as protein standards. The proteins were fixed in 10% TCA for 30 min, followed by 1% TCA for 10 min. The changes in isoelectric points (pI) of the applied proteins (recombinant proteins and proteins from the cell lysates) were examined. Both recombinant tick cystatins; isolated from insect cells and from *E. coli*, were applied.

### SDS-PAGE and western blotting

Protein samples (cystatin OmC2, lysates from differentiated MUTZ-3 cells with internalized cystatin OmC2) were separated on 12.5% pre-cast gels from Lonza using the Mini-PROTEAN 3 Cell system (Bio-Rad). Protein standards from Fermentas (PageRuler Prestained Protein Ladder) were applied. The separated proteins were stained with 0.1% PhastGel Blue R (Coomassie R 350 dye) from GE Healthcare or with 0.2% AgNO_3_ (Fluka). Selected bands were excised and analyzed using mass spectrometry or the proteins were transferred to PVDF membranes. The proteins from the SDS-PAGE or IEF gels were electrotransferred to PVDF membranes and immunolabeled with rabbit anti-cathepsin S pAb (Zavašnik-Bergant et al., 2005), goat anti-cathepsin S pAb (AF1183, R&D Systems), mouse anti-cathepsin C mAb (sc-74590, Santa Cruz Biotechnology), or mouse anti-β-actin antibody (A1978, Sigma-Aldrich). ECL Western blotting detection reagents (Amersham, GE Healthcare) were applied, and the imaging films (BIOMAX Light Film, Carestream) were developed (Konica Minolta SRX-101A). Alternatively, the bands were detected using the DAB chromogenic substrate (Sigma-Aldrich).

### Mass spectrometry (MS) of recombinant proteins

Selected bands were excised from the precast SDS-PAGE or IEF gels and prepared for mass spectrometric analysis as described (Sobotič et al., 2015). Briefly, the gel pieces were de-stained, reduced with 10 mM DTT and alkylated with 55 mM iodoacetamide in 25 mM ammonium bicarbonate buffer, pH 7.8. In-gel digestion was performed with sequencing grade modified porcine trypsin (Promega) at 37°C, overnight. The samples were subjected to peptide mass fingerprint analysis using a MALDI-TOF/TOF UltraFlextreme III mass spectrometer (Bruker), and the database was searched using Mascot software. To compare the non-labeled cystatin OmC2 and Alexa Fluor 488-labeled cystatin OmC2, the intact protein molecular weights were determined using MALDI-TOF/TOF UltraFlextreme III. For this purpose, a matrix mixture was prepared by mixing 2 μl of protein sample, 2 μl of 2% TFA in H_2_O and 2 μl of a 2,5-dihydroxyacetophenone matrix solution. The mixture was applied to a matrix target plate, allowed to dry at room temperature, and analyzed.

### Differentiation and maturation of cells

The human MUTZ-3 cell line was purchased from DSMZ (Germany). The cells were grown in α-MEM with 20% heat-inactivated FBS (PAA Laboratories-GE Healthcare Life Sciences), 1% Glutamax (Life Technologies) and 40 ng/ml GM-CSF (CellGro) as previously described (Nelissen et al., 2009; Song et al., 2015). The MUTZ-3 cells were differentiated to immature DC with 62.5 ng/ml GM-CSF, 100 ng/ml IL-4 (CellGro) and 2.5 ng/ml TNF-α (CellGro) for 4 days (Zavašnik-Bergant and Bergant Marušič, 2016). The viability of the cells was evaluated using Trypan Blue (Sigma-Aldrich). The differentiated MUTZ-3 cells were matured with 20 ng/ml LPS (Sigma-Aldrich) for 24 h. Alternatively, the MUTZ-3 cells were stimulated with LPS in the presence of tick cystatin OmC2 (0.4 or 0.8 μM). Cystatin solution was filtered through a 0.22 μM Durapore PVDF membrane/Millex GV filter unit (Millipore). In control experiments, sterile PBS was added instead of cystatin OmC2, and the cells were cultured for 24 h. In addition, immature DC were matured with LPS in the presence of 0.4 or 0.8 μM human cystatin C (Zavašnik-Bergant et al., 2005).

### Flow cytometry

The surface expression of MHC II and CD86 was followed in MUTZ-3 cells (differentiated and stimulated with LPS or cultured in the presence of recombinant tick cystatin OmC2 or human cystatin C). Both cystatins weren't labeled with Alexa Fluor 488. The cells were pre-incubated at 4°C to prevent non-specific internalization of the fluorescently labeled primary antibodies: anti-CD86 (clone FUN-1) conjugated to CY-CHROME and anti-MHC II (anti-HLA-DR, clone G46-6) conjugated to R-phycoerythrin (both from BD Biosciences-Pharmingen). A total of 5000 cells per sample were gated and evaluated for specific labeing using a FACSCalibur instrument (BD Biosciences) and BD CellQuest software (version 3.3). Differentiated MUTZ-3 cells treated with cystatin OmC2 and LPS were compared to non-treated differentiated MUTZ-3 cells.

### Immunocytochemistry, confocal, and electron microscopy

MUTZ-3 cells (non-differentiated, differentiated, activated with LPS, or cultured in the presence of 2 or 12 μM tick cystatin OmC2 for 1 or 3 h) were cytocentrifuged to glass slides coated with poly-L-lysine (Sigma Aldrich). The cells were fixed with 4% paraformaldehyde and permeabilized with 0.1% Triton X-100. The cells were then immunolabeled with rabbit anti-cathepsin S pAb, rabbit anti-human cystatin C pAb, sheep anti-cathepsin L (Zavašnik-Bergant et al., 2005), mouse anti-HLA-DR mAb (clone TÜ-36, BD Pharmingen), goat anti-cathepsin C pAb (clone T-17, Santa Cruz Biotechnology) or rabbit anti-His tag pAb (ab9108, Abcam). Highly cross-adsorbed goat anti-mouse IgG pAb, goat anti-rabbit IgG pAb, donkey-anti sheep IgG pAb, and donkey anti-goat IgG pAb, labeled either with Alexa Fluor 488 or with Alexa Fluor 546, obtained from Life Technologies-Molecular Probes were used as secondary antibodies. Controls in which the primary or secondary antibodies were omitted were included in these runs. Immunofluorescence microscopy of optical sections was performed by using a confocal laser scanning microscope Leica TCS SP5 X (Leica MicroSystems). The fluorophores were excited with selected lines from a tunable white light laser (460–670 nm) or a diode laser (405 nm). Leica Application Suite Advanced Fluorescence software (LAS AF, version 2.7.3.9723, Leica MicroSystems) was used for the image analysis. Transmission electron microscopy and immunogold labeling of cystatin C was performed as described (Zavašnik-Bergant and Bergant Marušič, 2016).

### Live cell imaging

Differentiated MUTZ-3 cells were cultured in the presence of fluorescently labeled tick cystatin OmC2 (2 μM) for 1 or 3 h. Internalization of cystatin OmC2 to acidic vesicles within the endocytic pathway of treated cells, i.e., to the late endosomes and lysosomes, was followed by using confocal microscopy and fluorescently labeled organelle markers. The LysoTracker Blue DND-22 (a marker for lysosomes), CellMask Orange plasma membrane stain, and CellLight Late Endosomes-RFP/Bac Mam 2.0 system (for the expression of Rab7a-RFP in late endosomes) were from Life Technologies-Molecular Probes. All commercial reagents were used according to the supplier's recommendations. Also, differentiated MUTZ-3 cells were pre-incubated for 30 min at 4°C to decrease their endocytic capacity. Then, cells were cultured with fluorescently labeled cystatin OmC2 at 4°C for 1 and 3 h. Cells cultured at 4°C were compared to the cells cultured at 37°C (and not pre-incubated at 4°C).

### Cell lysates

Differentiated MUTZ-3 cells (1 × 10^6^ cells/ml) were cultured in the presence of 2, 12, or 15 μM cystatin OmC2 for 1 or 3 h. The cells were pelleted, vigorously rinsed and resuspended in 0.1 M phosphate buffer with 1 mM EDTA (pH 6.0). The cells were sonicated on ice using a Branson Digital W-450 Sonifier (Branson, Danbury, CT). The non-soluble fraction was pelleted and removed by centrifugation at 16,000 g. The soluble fraction was aliquoted and stored at −80°C. The residual activity of the cysteine proteases in the treated cells was compared to the non-treated cells (control). In addition, lysates for analysis by mass spectrometry and Western blotting were prepared from cells that had been cultured in the presence of 12 μM non-labeled cystatin OmC2 (pull-down assay).

### Activity of cysteine proteases

The residual endoprotease and exoprotease activities were measured using the fluorogenic substrates Z-Phe-Arg-AMC and H-Gly-Phe-AMC (Bachem) as described (Mihelič et al., 2008; Poreba et al., 2014). The increases in the fluorescence signals (ex. 370 nm/em. 460 nm) were measured with Tecan Safire microplate reader. Samples were measured in triplicate and the differences in residual activity after 1 h and after 3 h were analyzed with Student's *t*-test. Prior to the *t*-test an assumption on the homogeneity of variances was tested by the Levene test. Cells cultured in the presence of cystatin OmC2 (two different times of incubation at 37°C, two fluorogenic substrates, and three concentrations of added inhibitor) were all compared to corresponding controls (non-treated cells). *P*-values of 0.05 or less were considered significant. Statistical analysis was performed using IBM SPSS Statistics 20.

### Pull-down assay

The isolation of the cell proteins bound to internalized cystatin OmC2 (not labeled with Alexa Fluor 488) was performed using immobilized-Ni affinity chromatography. Cell lysates were prepared from differentiated MUTZ-3 that had been cultured in the presence of 12 μM cystatin OmC2 for 1 or 3 h as described above. PBS was added to non-treated cells as a control. The lysates from the treated and non-treated cells were normalized to the same protein content (Bradford assay, Bio-Rad) and incubated with Ni-precharged Sepharose CL-6B (Roche). After 30 min of incubation at 4°C and rigorous shaking (200 rpm), the beads with the bound proteins were rinsed, mixed with SDS-PAGE loading buffer with DTT and boiled. The samples were centrifuged at 16,000 g, and the soluble fractions were subjected to electrophoresis. The separated proteins were stained with Coomassie dye and analyzed using mass spectrometry. Alternatively, the separated proteins were blotted to nitrocellulose membranes and labeled with anti-cathepsin C or anti-cathepsin S antibodies.

### Mass spectrometry of cell lysates

Selected comparable bands were cut from the precast SDS-PAGE gels and prepared as described (Sobotič et al., 2015). The extracted peptides were analyzed using an Orbitrap LTQ Velos mass spectrometer (Thermo Scientific, Waltham, MA, USA), coupled to a nanoLC HPLC unit (Proxeon, Thermo Scientific, Waltham, MA, USA). The peptides were loaded on a C_18_ trapping column (Proxeon EASY-Column^TM^) and separated on a C18 PicoFrit^TM^ AQUASIL analytical column (New Objective, Woburn, MA, USA). The mobile phase A solvent (0.1% formic acid) was used for loading and a 60 min gradient consisting of 5–40% of mobile phase B (100% acetonitrile, 0.1% formic acid) was used for peptide separation. The overall flow rate was 300 nl/min. The MS spectra were acquired in the Orbitrap analyser with a mass range 300–2,000 m/z and a resolution of 30,000. The MS/MS spectra were obtained by CID fragmentation (normalized collision energy at 35) of the nine most intense precursor ions from the full MS scan. Dynamic exclusion was enabled with repeat count of 1 and 60 s exclusion time. The database search and quantification by spectral counting were performed using the MaxQuant proteomics software (version 2.0.18), with the embedded Andromeda search engine (Cox and Mann, 2008; Cox et al., 2011). Searches were performed against the human IPI protein database v.385 (89,952 sequences, 36,291,020 residues), using the trypsin cleavage specificity with a maximum of 2 missed cleavages. Carbamidomethylation of cysteines was set as a static modification, while methionine oxidation and N-terminal acetylation were set as dynamic modifications. A reversed database search was performed and the false discovery rate (FDR) was set at 1% for peptide and protein identifications. Proteins with at least two identified peptides were considered to be positive identifications. Relative quantification was performed by spectral counting as described previously (Old et al., 2005).

## Results

### Characterization of cystatin OmC2

**Figure 1:**
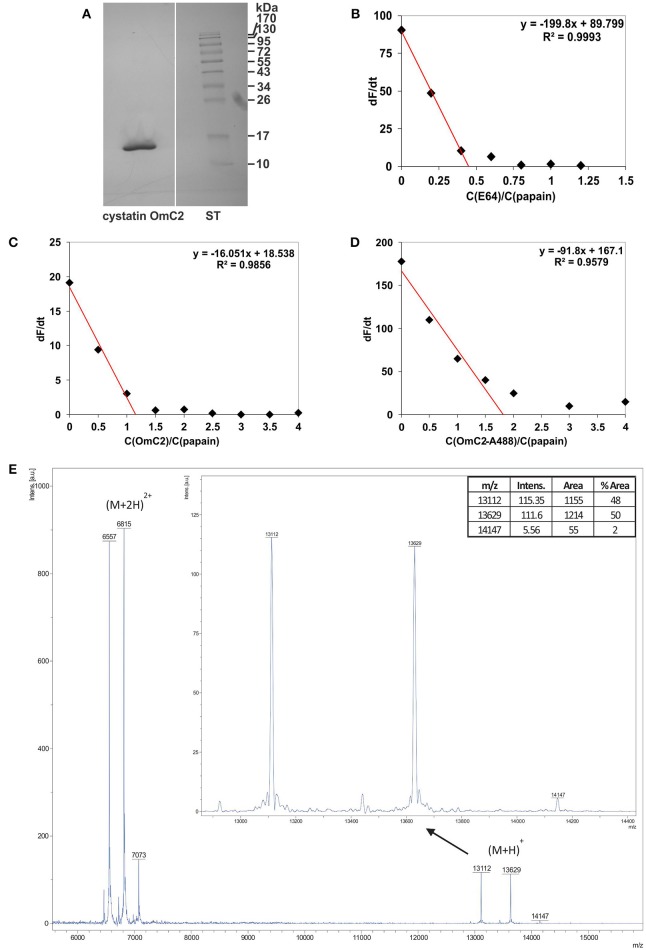
Characterization of cystatin OmC2 by **(A)** SDS-PAGE, **(B–D)** active site titration and **(E)** mass spectrometry. **(A)** The sample was stained with silver, ST—standards. **(B)** Active site titration of papain with inhibitor E64; dF/dt = 0 at a molar ratio of papain/E64 = 0.45. **(C)** Active site titration of papain with cystatin OmC2; dF/dt = 0 at a molar ratio of cystatin OmC2/papain = 1.16. **(D)** Active site titration of papain with cystatin OmC2 conjugated to Alexa Fluor 488; dF/dt = 0 at a molar ratio of cystatin OmC2-A488/papain = 1.82. **(E)** MALDI-TOF spectra of non-labeled cystatin OmC2 and cystatin OmC2 conjugated to Alexa Fluor 488. Singly charged cystatin OmC2 peaks corresponding to different modification levels ((M+H)^+^): the peak at 13112 Da represents unmodified cystatin OmC2, the peak at 13629 Da represents N-terminally labeled cystatin OmC2, and the peak at 14147 Da represents cystatin OmC2 labeled at its N-terminal and a Lys side chain. The areas of singly charged peaks (enlarged image) were used for relative quantification of labeling efficiency. Doubly charged peaks ((M+2H)^2+^) are also present.

Tick cystatin OmC2 was prepared in insect cells as a recombinant protein with oligo His-tag added to its C-terminus (Salát et al., 2010). Recombinant cystatin OmC2 from insect cells, previously checked for endotoxin contamination, was used in the majority of described experiments. Recombinant cystatin OmC2 from insect cells was preferentially applied for two reasons; (1) it was added to the immune cells later on, and (2) it was expressed with His-tag at the C-terminal end of cystatin molecule, i.e., at the part which does not participate in binding to the active site of targeted proteases (lysosomal cathepsins). The isolated protein migrated as a single band at approximately 13 kDa (Figure 1A). The inhibitor was 88% active as determined by active site titration with papain (Figures 1B,C) whereas the activity of cystatin OmC2 conjugated to the Alexa Fluor 488 fluorophore was 55% (Figure 1D). Furthermore, the molecular weights of non-labeled and fluorescently labeled cystatin OmC2 were confirmed by mass spectrometry to verify the fluorophore binding (Figure 1E). Three singly charged peaks were observed. The first peak corresponded to the non-labeled cystatin OmC2 with a mass of 13112 Da, which is in agreement with its theoretical Mw of 13115 Da. Two additional species were determined, the first of which corresponded to cystatin OmC2 that was N-terminally conjugated with Alexa Fluor 488 (13629 Da), and the second was a minor species that corresponded to the additional labeling of a lysine side chain (14147 Da). According to the areas of the peaks corresponding to each species, nearly 50% of cystatin OmC2 was N-terminally conjugated and only a minor part was additionally labeled at the lysine side chain (< 2%). Additional peaks were observed for doubly charged species at 6557, 6815, and 7073 Da. No other bands or additional peaks that would have indicated the presence of impurities were observed on a silver-stained SDS-PAGE gel (Figure 1A) or in the MS spectra (Figure 1E). Both non-labeled and fluorescently labeled cystatin OmC2 were used in the subsequent studies with cells.

### Cystatin OmC2 decreases surface expression of MHC II and CD86 in LPS-stimulated cells

**Figure 2:**
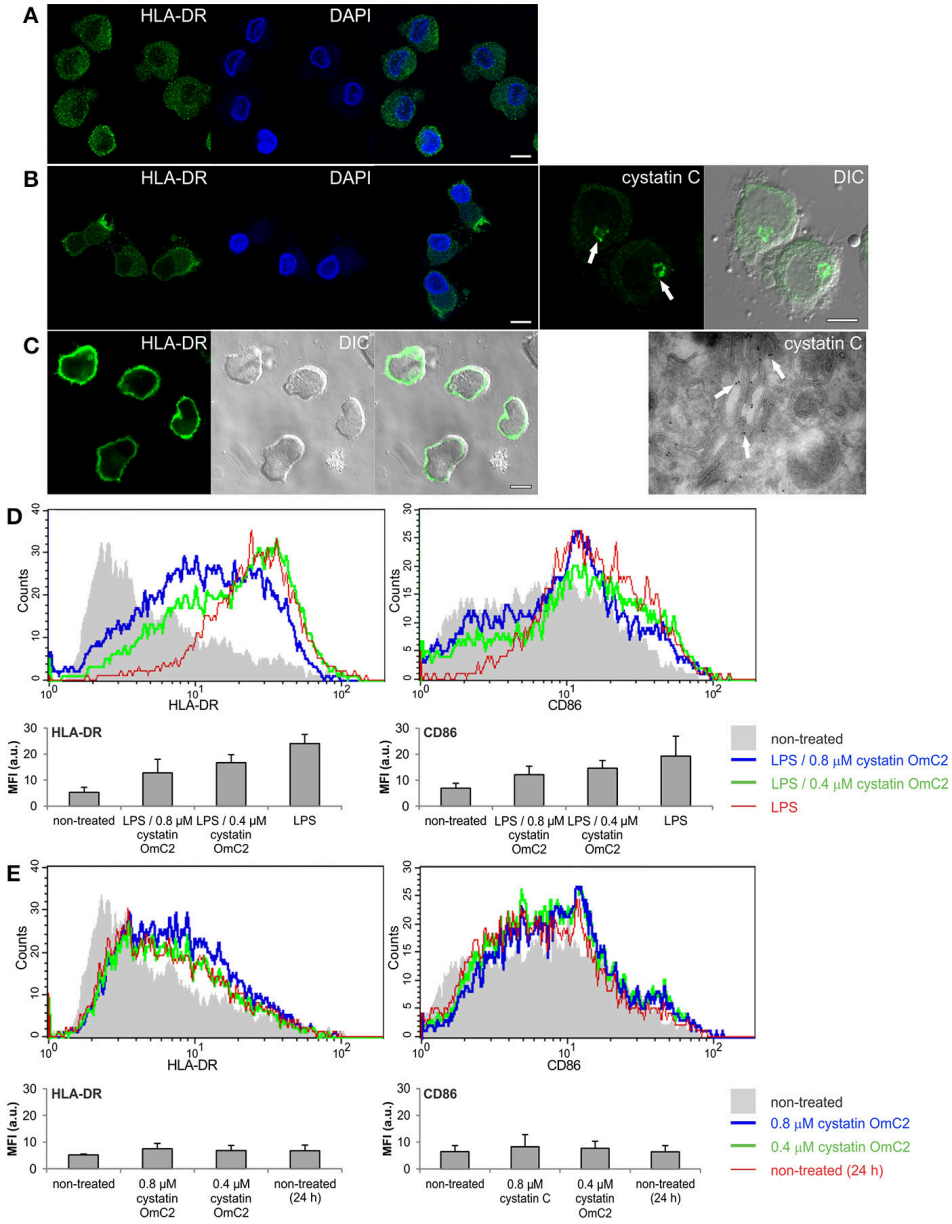
Effect of tick cystatin OmC2 on the expression of MHC II and CD86. The confocal images show MHC II (HLA-DR) in **(A)** non-differentiated MUTZ-3 cells, **(B)** differentiated MUTZ-3 cells, and **(C)** differentiated MUTZ-3 cells stimulated with LPS. The localization of endogenous cystatin C in the differentiated MUTZ-3 cells is shown in **(B)**. Bars: 10 μm. **(D)** LPS-induced maturation of differentiated MUTZ-3 cells in the presence of cystatin OmC2 is compared to non-treated cells (no added cystatin OmC2). **(E)** The histograms show HLA-DR and CD86 in the differentiated MUTZ-3 grown in the presence of cystatin OmC2 (no maturation with LPS). **(D,E)** The shadowed histograms represent the non-treated cells (no added cystatin OmC2 and no maturation with LPS) at the beginning of the experiment. A representative analysis of three independent biological replicates is shown. Mean fluorescence intensities (MFI) of labeled cell populations (geometric means) are shown in bar graphs.

MUTZ-3 cells (Figure 2A) that had been differentiated with IL-4 were used as a model of immature DC. An increase in the surface expression of MHC II (Figure 2B) and a high content of endogenous cystatin C in the Golgi apparatus (Figure 2B, arrows), which is another characteristic of human immature DC when they are differentiated *in vitro* (Zavašnik-Bergant et al., 2005), were confirmed in the differentiated MUTZ-3 cells (Figure 2B). In addition to confocal imaging, an increased immunogold labeling of cystatin C in Golgi cisternae was observed in differentiated MUTZ-3 cells (Figure 2B, TEM micrograph, arrows). MHC II was further increased in these cells following a 24-h maturation with LPS (Figure 2C). The increases in the expression of MHC II and CD86 that were associated with the maturation induced by LPS (red histograms, bar graphs, Figure 2D) were diminished when cystatin OmC2 was added to the culture medium (green and blue histograms, Figure 2D). In contrast, cystatin C, which is a human cystatin type 2 homolog that is endogenously expressed (Figure 2B) and secreted by human DC (Zavašnik-Bergant et al., 2005), did not notably change the surface expression of CD86 when it was added to the immature DC together with LPS at the same concentrations (0.4 or 0.8 μM) as the tick cystatin OmC2 (Supplementary Figure 2A), or added to the culture medium of the immature DC in the absence of LPS (Supplementary Figure 2B). Furthermore, pre-incubation of the immature DC with cystatin OmC2 in the absence of stimulation with LPS did not noticeably change the surface expression of MHC II or CD86 (Figure 2E). If, supposedly, the applied recombinant cystatin OmC2 had been “contaminated” with endotoxins, an increased labeling of surface HLA-DR and CD86 might have been observed also in cells shown in Figure 2E (cells not stimulated with LPS).

### Cystatin OmC2 is internalized via the endocytic pathway of differentiated MUTZ-3 cells

The Alexa Fluor 488-conjugated cystatin OmC2 was efficiently separated from the unbound Alexa Fluor 488 dye after three steps of purification. After the second dialysis and membrane filtration, the fluorescence of the unreacted dye in the filtrate was low (1125 a.u.) compared to the fluorescence measured in fraction with the labeled cystatin (25900 a.u.) (Supplementary Figure 3A). Compared to the preincubation of cells with the labeled cystatin OmC2 (Supplementary Figure 3C), no pronounced fluorescence was observed when cells were pre-incubated (under the same conditions) with the equivalent volume of the filtrate after the last purification step (Supplementary Figure 3D). The filtrate contained only the remaining unbound dye but not the conjugated protein. We concluded that the conjugated cystatin OmC2 was applicable and it was therefore used for the co-localization study with immunolabeled cathepsin S and cathepsin C.

Differentiated MUTZ-3 cells showed high internalization ability of fluorescently labeled cystatin OmC2 at 37°C (Supplementary Figure 4). Increased fluorescence was observed in Rab7a-positive vesicles after 1 and 3 h (Supplementary Figures 4C,D). On the contrary, the internalization of fluorescently labeled cystatin OmC2 was almost completely abolished in cells that were cultured at 4°C for 1 h and 3 h (Supplementary Figures 4A,B). In addition, a clear distinction between the non-viable cell (being fluorescent due to the loss of its membrane integrity) and other viable cells (without significant intracellular fluorescence) can be seen in Supplementary Figure 4A. The diffused green fluorescence can be observed outside the cells because the fluorescently labeled cystatin OmC2 was not removed from the culture medium before confocal imaging.

**Figure 3:**
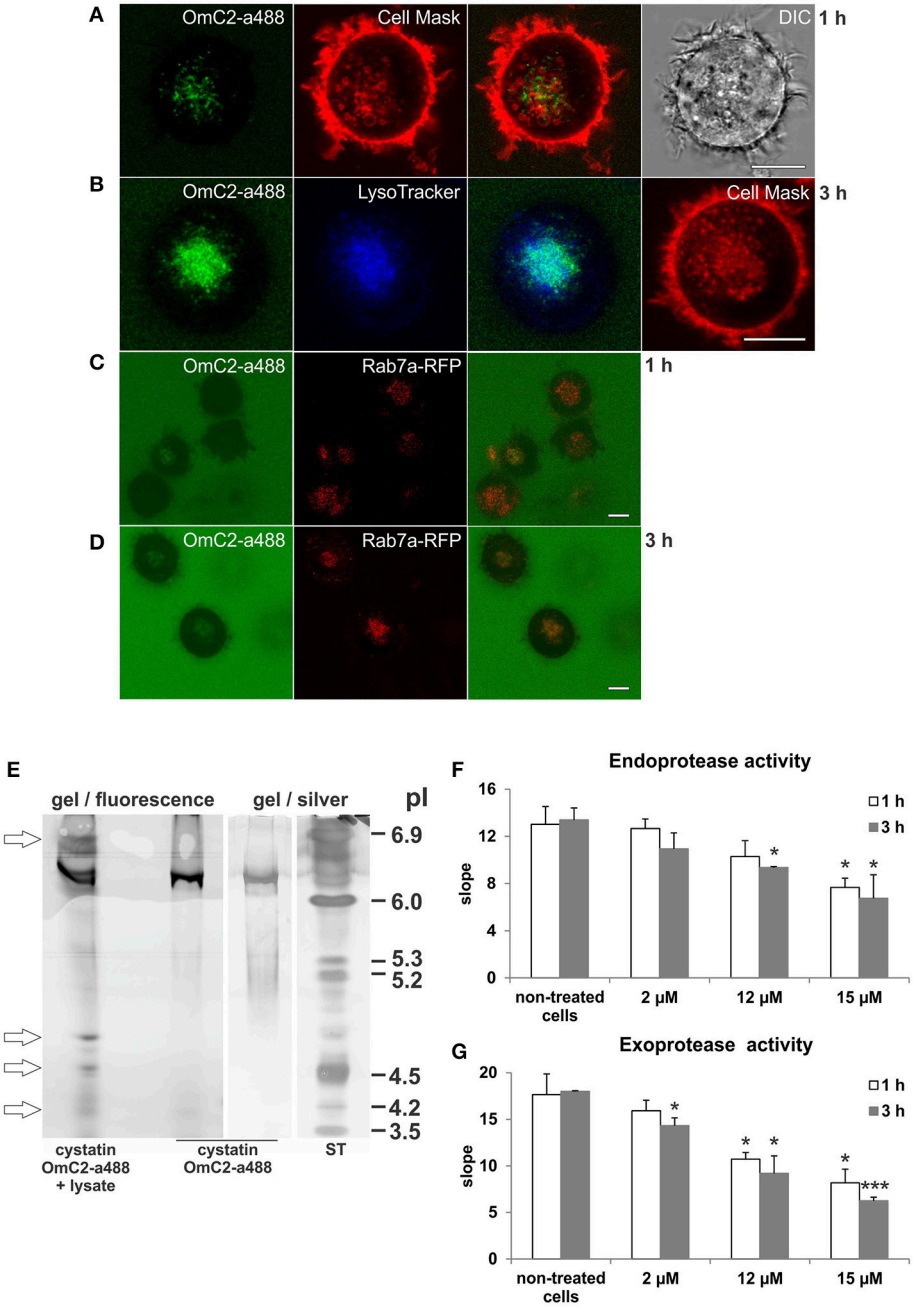
Localization of internalized cystatin OmC2 and the activity of cysteine proteases in treated differentiated MUTZ-3 cells. Cystatin OmC2 labeled with Alexa Fluor 488 **(A−D)**, LysoTracker Blue DND-22 **(B)**, CellMask Orange plasma membrane stain **(A,B)**, and Rab7a-RFP **(C,D)** are shown in viable cells. Bars: 10 μm. **(E)** IEF (pH 3–7) of the proteins from cells that were cultured in the presence of labeled cystatin OmC2 for 3 h. The pI-values of the additional fluorescent bands in the lysate are denoted (arrows) and compared to the pI of the fluorescently labeled cystatin OmC2, which was also stained with silver, at pI 6.3. ST—standards. **(F,G)** The endoprotease (Z-Phe-Arg-AMC) and exoprotease activities (H-Gly-Phe-AMC) in the lysates from cells that were cultured with non-labeled cystatin OmC2 (2, 12 or 15 μM) for 1 or 3 h. The samples were measured in triplicate, and the average values ± *SD* are shown. Cell treated with cystatin OmC2 were compared to non-treated cells after 1 h or after 3 h (*t*-test, ^*^P < 0.05, ^***^P < 0.001).

The plasma membrane of the cells was stained with CellMask Orange, and the newly formed endocytic vesicles of the cells that were cultured in the presence of cystatin OmC2 were observed under the confocal microscope (Figures 3A,B). Following its internalization, cystatin OmC2 was found present in vesicles formed from the labeled plasma membrane (Figure 3A). This confirmed that its internalization from the culture medium to the endocytic pathway of the treated cells was successful. After 1 h, cystatin OmC2 was already localized in the Rab7a-positive late endosomes (Figure 3C). The uptake of cystatin OmC2 was not homogenous within the population of the treated cells, but the fluorescence associated with cystatin OmC2 clearly increased during the prolonged 3 h incubation (Figure 3D). After this time, more than 80% of cells showed an intense perinuclear staining of the internalized cystatin OmC2.

In addition, the predicted binding of internalized fluorescently labeled cystatin OmC2 to target proteins in differentiated MUTZ-3 cells was further indicated by the changes of isoelectric point (pI) of cystatin OmC2 that were observed in the IEF gels under non-denaturating conditions in the range of pH 3–7 (Figure 3E). Prior to its internalization, fluorescently labeled cystatin OmC2 was detected at pI 6.3 (Figure 3E). In contrast, in the lysates prepared from cells cultured in the presence of cystatin OmC2, additional fluorescent bands at 4.2, 4.5, 4.7, and 6.8 were observed (Figure 3E, arrows). The multiple fluorescent bands in the lysate, in addition to the one at pI 6.3, indicated that internalized cystatin OmC2 was bound to various cell constituents. No fluorescent bands were observed in lysates prepared from cells cultured in the absence of the labeled cystatin OmC2.

### Cystatin OmC2 decreases the activity of cysteine proteases in differentiated MUTZ-3 cells

The endoprotease activity toward Z-Phe-Arg-AMC (Figure 3F) and the exoprotease activity toward H-Gly-Phe-AMC (Figure 3G) were both decreased in the lysates from the immature DC that were cultured in the presence of cystatin OmC2 for 1 or 3 h. Compared to the non-treated cells, the endoprotease activity after 3 h was diminished by 18% in the cells treated with 2 μM cystatin OmC2 and by 30% in cells treated with 12 μM cystatin OmC2. The exoprotease activity was diminished more, i.e., by 26% at 2 μM cystatin OmC2 and by 49% at 12 μM cystatin OmC2 when the inhibitor was added to the culture medium of differentiated MUTZ-3 cells. However, even at the highest concentration of cystatin OmC2 applied (15 μM), the endoprotease and exoprotease activities were not completely blocked after 3 h; the first was diminished by 50% and the second by 65%. The addition of cystatin OmC2 did not noticeably change the viability of the treated cells after the 3-h incubation compared to the non-treated cells (Supplementary Table 1). Significant differences in protease activity between the non-treated cells (control) and the cells treated with cystatin OmC2 at different experimental conditions are denoted in Figures 3F,G.

### Cystatin OmC2 forms complexes with recombinant cathepsins S and C

**Figure 4:**
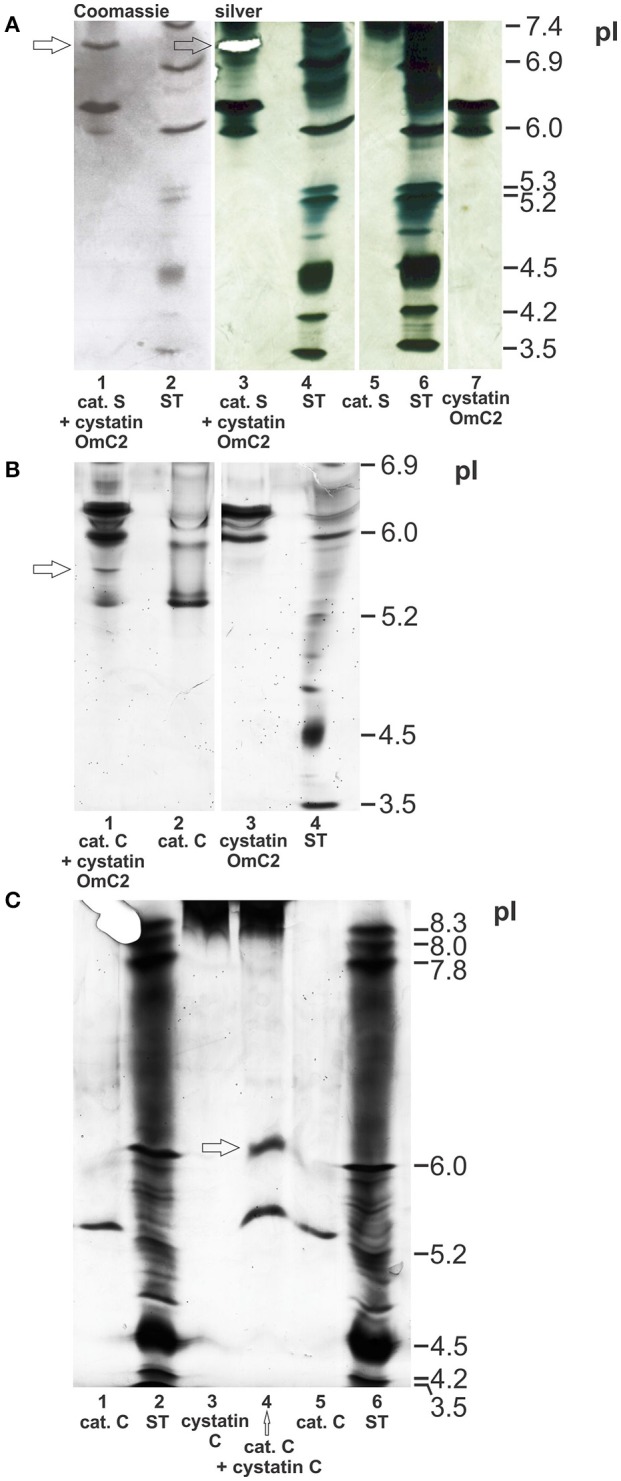
Formation of complexes of cystatin OmC2 with cathepsin S and of cystatin OmC2 with cathepsin C. IEF was performed at pH 3–7 **(A,B)** and pH 3–10 **(C)** under native conditions. The cathepsins were pre-incubated with cystatin OmC2 **(A,B)** or with cystatin C **(C)** prior to IEF. The samples (all recombinant proteins) are: cystatin OmC2 **(A,B)**, cathepsin S **(A)**, cathepsin C **(B,C)** and cystatin C **(C)**. The cystatin OmC2/cathepsin S complex is indicated at pI 7.1, the cystatin OmC2/cathepsin C complex at pI 5.6, and the cystatin C/cathepsin C complex at pI 6.2. The proteins were stained with silver **(A–C)** and Coomassie dye **(A)**. **(A)** The excised bands, analyzed by peptide mass fingerprint (A, lane 3 and B, lane 1) are indicated (white mask, arrows). ST, standards.

The possible formation of complexes of cystatin OmC2 with the selected lysosomal endoprotease cathepsin S, and of cystatin OmC2 with the exoprotease cathepsin C, was verified using IEF (pH 3–7) under native conditions. The observed changes in the pI's of cathepsin S and of cathepsin C, both pre-incubated with cystatin OmC2, were compared to those of the non-treated cathepsins (Figure 4). The predicted pI for cystatin OmC2 (Salát et al., 2010) was confirmed to be 6.3 (Figures 4A,B). Cystatin OmC2 is a secretory type 2 cystatin. We presume that another band around pI 6.0 (Figure 4A, lane 7), in addition to the band at pI 6.3, represents the different glycosylation of recombinant cystatin OmC2 molecules expressed in insect cells. According to the cystatin OmC2 sequence, O-glycosylation at four Ser and Thr residues, respectively, has been predicted using NetOGlyc 4.0 software. A pI above 8.0 was determined for human cystatin C, which is an endogenous type 2 cystatin inhibitor of cysteine proteases (Figure 4C).

The bands representing cathepsin S (without cystatin OmC2) were observed at pI 7.4 or higher (Figure 4A, Supplementary Figure 1). Multiple bands were observed due to the different glycosylation states of the recombinant cathepsin S. The recombinant cathepsin C was observed at pI 5.3 (stronger band) and pI 6.0 (Figures 4B,C). Pre-incubation of the cysteine proteases with tick cystatin OmC2 resulted in the appearance of additional bands: an additional band at pI 7.1 was observed in the case of cathepsin S (Figure 4A, arrow) and one at pI 5.6 in the case of cathepsin C (Figure 4B, arrow).

A band representing a complex between cathepsin S and the tick cystatin OmC2 that was expressed in *E. coli* was also observed at pI 7.1 (Supplementary Figure 1). In addition, a new band indicating the formation of a cathepsin C/human cystatin C complex was observed at pI 6.2 (Figure 4C). Cystatin C was observed as a thick diffused band above pI 8.3 (Figure 4C, lanes 3 and 4). Applied cystatin C is a basic protein with a very high pI, so it didn't travel far in an electric field toward the other electrode as did travel other proteins with lower pI-values. But, regardless of the high pI of cystatin C, the distinction between the pI-value of cystatin C bound to cathepsin C (at pH 6.2) and the pI-value of cathepsin C alone (at pH 5.3) can be clearly drawn (Figure 4C, lane 4).

Credible protease/cystatin complexes of both cathepsin S and cathepsin C with cystatin OmC2 (Figures 4A,B) were identified in the excised bands by mass spectrometry (Supplementary Table 2). The identification of peptides that corresponded to the proteins under study in the single band excised from the IEF gels further confirmed the formation of complexes between the two studied proteins. In the case of the complex of cathepsin S with cystatin OmC2, 5 peptides from cathepsin S (15% sequence coverage) and 2 peptides from cystatin OmC2 (28% sequence coverage) were identified. In the case of the complex of cathepsin C with cystatin OmC2, 2 peptides from cathepsin C (4% sequence coverage) and 7 peptides from cystatin OmC2 (64% sequence coverage) were identified.

### Cystatin OmC2 colocalizes with cathepsins S and C in differentiated MUTZ-3 cells

**Figure 5:**
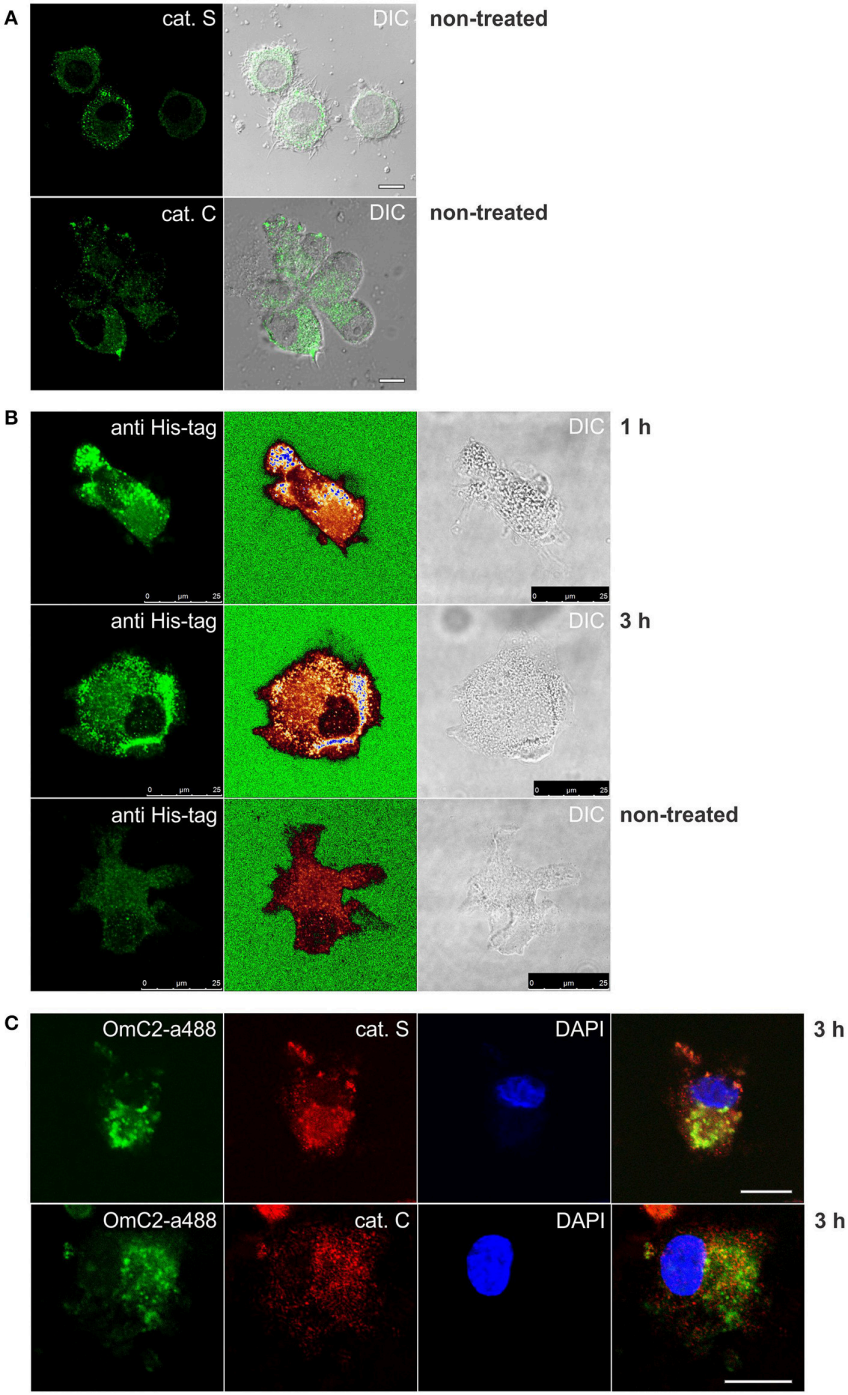
Immunocytochemistry of differentiated MUTZ-3 cells that were cultured in the presence of cystatin OmC2 for 1 and 3 h. The confocal images show **(A)** immunolabeled cathepsins in non-treated cells, and **(C)** their colocalization with Alexa Fluor 488-labeled cystatin OmC2 in treated cells. **(B)** Cells with internalized cystatin OmC2 (without fluorochrome) were labeled with an anti-His tag antibody. The intensity of the fluorescence signal is represented with a rainbow color palette (green–the lowest intensity, blue–the highest intensity). Non-treated cells were used as a control. Bars: 10 μm **(A,C)** and 25 μm **(B)**.

The formation of complexes of cystatin OmC2 with recombinant cathepsin S, and of cystatin OmC2 with recombinant cathepsin C (Figure 4), indicated that similar complexes could form between internalized tick cystatin and its target proteases in immune cells if the cystatin OmC2 colocalized with the proteases inside the same cell compartments (late endosomes and lysosomes). We further hypothesized that those complexes would be stable enough to be isolated from the treated cells. Confocal micrographs confirmed that the fluorescently labeled cystatin OmC2 accessed the acidic vesicles (lysosomes) that were labeled with Lysotracker (Figure 3B) and have a high content of lysosomal cysteine proteases (Figure 5A). Among these vesicles, the signals for cathepsin C and cathepsin S were found to colocalize with the internalized cystatin OmC2 (Figure 5C). The vesicular staining of the internalized non-labeled cystatin OmC2 after 1 and 3 h was confirmed using an anti-His tag antibody (Figure 5B). In addition, comparable vesicular patterns of internalized fluorescently labeled cystatin OmC2 (green fluorescence) and cystatin OmC2 labeled with an anti-His tag antibody (red fluorescence) were confirmed in the same cells after a 3 h-incubation (Supplementary Figure 5). Concerning the vesicles with red fluorescence only (Supplementary Figure 5), they represent the internalized cystatin OmC2, not labeled with Alexa Fluor 488 prior to the experiment.

### Cystatin OmC2 binds to cysteine cathepsins in differentiated MUTZ-3 cells

**Figure 6:**
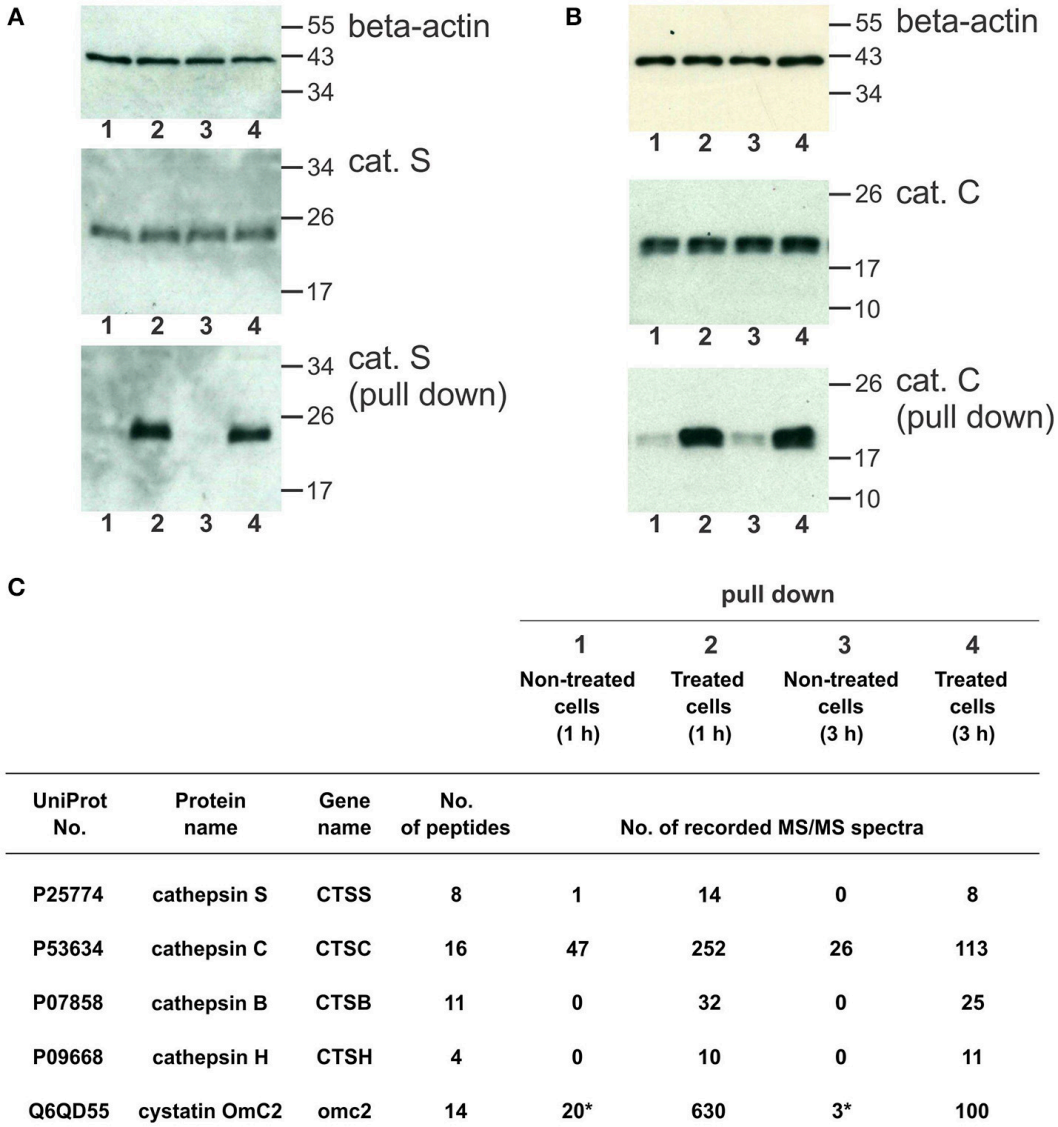
Pull-down of internalized cystatin OmC2 and endogenous cathepsins S and C from differentiated MUTZ-3 cells. **(A,B)** Western blotting of cathepsin S and cathepsin C in lysates and pull-down fractions from non-treated (lanes 1 and 3) and treated cells (lane 2 and 4). A total of 40 μg of total protein from lysates was added per well and beta-actin was used as a loading control. For the pull-down fractions, equal volumes (30 μl) per well were used. **(C)** The cathepsins in the pull-down fractions were identified using LC-MS/MS analysis, and the numbers of recorded MS/MS spectra are indicated. (^*^) A carry-over from an inhibitor-treated sample, analyzed as the preceding sample.

The cystatin OmC2 that was internalized after 1 and 3 h did not change the processing of cathepsin S (24 kDa) or cathepsin C (21 kDa) in the treated cells (lanes 2 and 4) compared to the cells grown in the culture medium without cystatin OmC2 (controls in lanes 1 and 3; Figures 6A,B).

Affinity binding of the oligo His-tag of the recombinant cystatin OmC2 enabled purification of the targeted cellular proteins that were colocalized and successfully associated with the internalized cystatin. Hence, lysosomal proteases bound to the internalized cystatin OmC2 were isolated from the lysates of the differentiated MUTZ-3 cells. As shown using Western blotting, both, cathepsin S (Figure 6A) and cathepsin C (Figure 6B) were found to be enriched in the pull-down fractions from the treated cells (lanes 2 and 4) compared to the cells grown in the absence of cystatin OmC2 (controls in lanes 1 and 3).

Mass spectrometry was applied to further determine whether cathepsins S and C were among proteins targeted by cystatin OmC2. Several cysteine cathepsins were identified in the pull-down fractions from cells after 1 or 3 h (Figure 6C, Supplementary Table 3). Quantification by spectral counting showed that cathepsins B, C, H, and S were enriched in samples from the cells that were cultured in the presence of cystatin OmC2. All four cathepsins showed greater than five-fold increases in the spectral counts compared to the control cells that were not treated with cystatin OmC2, which is significant according to the criteria generally accepted for spectral counting (Old et al., 2005). Further, cathepsins B, H, and S were completely absent from samples of the non-treated cells, which additionally confirmed the relevance of the cystatin OmC2/cathepsin interaction for the enrichment. The same cathepsins (B, C, H, and S) were identified after 1 h and after 3 h of incubation of cells with cystatin OmC2. In contrast, following pre-treatment of cells with cystatin OmC2, cathepsins L and X were not significantly increased (Supplementary Table 3). Cystatin OmC2 was also highly enriched in the samples from the treated cells (Figure 6C). A small amount of cystatin OmC2 was identified in a control sample (Figure 6C) but was considered to be the result of a carry-over from the previous inhibitor-treated sample.

## Discussion

In haematophagous ectoparasites such as ticks, salivary modulators of the host DC have presumably evolved to suppress the host immune response to facilitate blood feeding (reviewed in Kovár, 2004; Schwarz et al., 2012; Kazimírová and Štibrániová, 2013). For those species that are vectors of pathogens, such molecules could also create a permissive environment for pathogen transmission (Preston et al., 2013). DC in the host skin participate in the development of the immune response against various species of bacterial pathogens such as *Borrelia* that are transmitted during the bite of a tick (Slámová et al., 2011; Lieskovská et al., 2015a,b). However, tick saliva inhibits the differentiation and maturation of DC, and subsequently modulates their immune stimulatory functions (Cavassani et al., 2005). Among the proteins that comprise the tick sialome, small secretory protease inhibitors similar to human endogenous cystatins have also been proposed to have a role in modulating the production of cytokines secreted from immune cells due to tick feeding (Sá-Nunes et al., 2009; Schwarz et al., 2012).

The salivary cystatin OmC2 from *Ornithodoros moubata* was evaluated as a model tick type 2 cystatin. This protein is homologous to human cystatin C, which is a member of family I25 of inhibitors of papain-like cysteine proteases [MEROPS database (Rawlings et al., 2016)]. Endogenous cystatin C is increased in monocyte-derived immature DC, but its high content in Golgi, the absence of significant colocalization with lysosomal cathepsins S, L, and H in the late endosomes and lysosomes, as well as an intensive secretion of cystatin C that is dependent on differentiation and maturation status of DC all indicate that the endogenous cystatin C does not directly modulate the activity of the endogenous cysteine proteases in DC (Zavašnik-Bergant et al., 2005; Zavašnik-Bergant, 2008). This conclusion contrasts with the report of Pierre and Mellman (1998) and the evidence that an exogenous cystatin homolog from *Brugia malayi*, Bm-CPI-2, inhibits the MHC II-restricted antigen processing in treated cells (Manoury et al., 2001).

On the basis of its similarity to human cystatin C, salivary tick cystatin OmC2 could be a potent immunomodulatory molecule that could mimic and add to the inhibitory function of its vertebrate homolog during the tick feeding and the transmission of tick-borne pathogens. In the present study, we have investigated the effect of cystatin OmC2 when it was internalized to the endocytic pathway of human immature DC, i.e., to late endosomes and lysosomes. Specifically, we asked whether the exogenous tick cystatin persisted in the acidic vesicles in contrast to endogenous cystatin C, and whether it changed the cell proteolytic potential and possibly also other DC characteristics. Cystatin OmC2 was tested for its abilities to inhibit the lysosomal cysteine proteases in DC prior to their maturation and to alter the surface expression of CD86 during the subsequent DC maturation, which could therefore indicate a possible function independent of its inhibitory activity.

The human acute myeloid leukemia cell line MUTZ-3 has been used as a model of dermal DC in human skin. Immature DC were generated from MUTZ-3 cells, previously differentiated *in vitro* with IL-4 (Song et al., 2015). Immature DC constantly sample the surrounding environment and endocytosis is a functional feature of these cells, which decreases with their maturation. As published by Larsson et al. (2006), the uptake of FITC-dextrane by mature MUTZ-3-derived DC, matured with either LPS or cocktail of proinflammatory cytokines, was reduced compared to immature MUTZ-3-derived DC. We confirmed that following the internalization of cystatin OmC2 by DC, it was extensively translocated to proteolytically active compartments, i.e., to the Rab7a-positive late endosomes (Guerra and Bucci, 2016), in which antigen processing and binding to MHC II take place (Blum et al., 2013), and to lysosomes, which are highly enriched with the active lysosomal cathepsins that are crucial for proteolytic degradation of the lysosome cargo. Further, the regulation of lysosomal degradation pathways is essential to maintain cellular homeostasis (Huber and Teis, 2016).

Cystatin OmC2 inhibits recombinant lysosomal cathepsins with K_*i*_-values in the nanomolar range, whereas the legumain/asparaginyl endopeptidase (AEP) from family C13 (Rawlings et al., 2016) is not inhibited (Grunclová et al., 2006). We demonstrated that both non-labeled and fluorescently labeled cystatin OmC2 were successfully internalized by the immature DC. The reduced but not completely abolished endoprotease and exoprotease activities of the cysteine proteases, and the confirmed colocalization demonstrated that the targeted proteases (including the lysosomal cathepsins S and C) were accessed by cystatin OmC2. The actual concentration of internalized cystatin OmC2 in particular subcellular compartments could not be measured. However, the inhibition of cysteine protease activity in DC was dependent on the concentration of applied cystatin OmC2 and was consistent with the increase of fluorescence signal from internalized inhibitor after the prolonged 3-h incubation. We did not observe that the added cystatin OmC2 vastly changed the viability of treated cells. To make DC incapable of an appropriate response to the tick antigens, it seems likely that the activity of targeted cysteine proteases in DC would be obstructed but not completely blocked by the incoming exogenous tick inhibitor.

We don't have quantitative data on the concentration of cystatin OmC2 in the tick saliva. In the paper by Grunclová et al. (2006) rough estimation of the amount of cystatin OmC2 in the salivary gland extract was reported (it represented 0.2−0.5% of the total protein content). In the paper by Salát et al. (2010) saliva collected from *O. moubata* adult females was subjected to proteomic analysis to directly confirm the presence of cystatin OmC2. The applied LC-MS/MS strategy was based on enzymatic digestion of a complex protein mixture and MS/MS peptide sequencing. The analysis provided 53% peptide coverage of the cystatin OmC2 sequence and confirmed that OmC2 is secreted in the saliva of *O. moubata*. Quantitative measurement of cystatin OmC2 concentration in secreted saliva is also problematic due to the small amount of obtained material from one adult tick (about 1 μl). Likely, the concentration used in *in vitro* studies is higher than the concentration of cystatin OmC2 in saliva. Then again, the saliva is injected directly into the tissue and the local concentration of its components may be quite high around the cells in that tissue. Though the tick may secrete only small amounts of cystatin OmC2, its inhibitory potency may be adequate to achieve inhibition of the targeted proteases.

Inhibitory activity of cystatin OmC2 has been determined in enzymatic assays with the recombinant mammalian cathepsins (Salát et al., 2010) at their optimal acidic pH, buffer composition and the absence of other proteases. Our first hypothesis was that cystatin OmC2 would preferentially bind to lysosomal cathepsins with endoprotease activity if successfully internalized to MUTZ-3 cells (and therefore affect their proteolytic activity). With respect to the published data, reported IC50 values (determined in assays with recombinant cathepsins) were in favor of that hypothesis. IC50 values of cystatin OmC2 were higher for cathepsin B (8.8 nM), cathepsin H (1.2 nM) or cathepsin C (1.1 nM), all of them exoproteases, whereas IC50 values of cystatin OmC2 were around 0.15 nM for reported endoproteases (papain, cathepsin S, and cathepsin L) (Salát et al., 2010), Thus, we anticipated more vigorous interaction between endoproteases and cystatin OmC2 also in differentiated MUTZ-3 cells.

The formation of complexes of the cystatin OmC2 with the recombinant cathepsin S and of cystatin OmC2 with the recombinant cathepsin C was verified by using IEF under native conditions, thus mimicking the conditions inside the DC endocytic pathway. Further, following the pre-treatment of the immature DC with cystatin OmC2, cathepsins C, B, and H were isolated from cell lysates together with internalized cystatin OmC2. This was also observed for cathepsin S but not for cathepsins L or X, even though cathepsin X is similar to cathepsin B and is also present in human DC (Obermajer et al., 2008). Cystatin OmC2 is a potent inhibitor of recombinant human cathepsins S and L with similar IC_50_ values (Salát et al., 2010). Conversely, a significant increase (over fivefold and more) in spectral counts compared to the control (non-treated cells) was confirmed only for cathepsin S but not cathepsin L (mass spectrometry analysis, Figure 6C), although both cathepsins are active in DC (Lennon-Duménil et al., 2002) and are present in differentiated MUTZ-3 cells (Figure 5, Supplementary Figure 1C). However, the vesicular staining of immunolabeled cathepsin L (Supplementary Figure 1C) was relatively weak compared to that for cathepsin S or to cathepsin C. The absence of cathepsin L among the proteins isolated in association with cystatin OmC2 indicates that the formation of this complex, although confirmed between cathepsin L and cystatin OmC2 as recombinant proteins (Supplementary Figure 1), might not occur in the treated DC.

Another possibility would be that cathepsin L/cystatin OmC2 complex, while transiently formed inside cells, was not stable enough to be isolated by affinity chromatography. By analogy, the p41 fragment from the p41 invariant chain, which is a chaperone to MHC II and an endogenous thyropin inhibitor of the cysteine proteases in APC, was shown to act as a chaperone of cathepsin L (Lennon-Duménil et al., 2001). By transiently binding to cathepsin L, this thyropin inhibitor helps to maintain a pool of the mature enzyme in the MHC II-loading compartments in mouse bone-marrow derived APC (Lennon-Duménil et al., 2001).

The inhibition of the endoproteases cathepsins S and L by cystatin OmC2 is comparable to that of sialostatin L, another salivary type 2 cystatin from the hard tick *Ixodes scapularis* (Salát et al., 2010). However, the inhibition of the exoprotease activity of cathepsins B, C, H by cystatin OmC2 is much more effective than that of sialostatin L (Salát et al., 2010); cystatin OmC2 inhibits cathepsin C with K_i_ = 0.19 nM (Grunclová et al., 2006). Our results demonstrated that substantial amounts of the analyzed cathepsins C, B, and H were present in the pull-down fractions from treated cells, which indicated that all of them, in addition to cathepsin S, bind to the internalized cystatin OmC2 (Figure 6C).

To summarize, we have shown that all three exoproteases (cathepsins C, B, and H) were increased in pull-down fractions from the cells with internalized cystatin OmC2, whereas only cathepsin S (an endoprotease) was increased when compared to non-treated cells. Neither cathepsin L nor other endoproteases were increased. Among the significantly increased proteases in pull-down fractions, we have focused on the two of them; on cathepsin S due to its known involvement in antigen processing and presentation in DC. Second, we have focused on cathepsin C, a dipeptidyl peptidase, often included in proteolytic cascades, but rarely studied in DC (Ishri et al., 2004; Hamilton et al., 2008).

Cysteine cathepsins are optimally active at slightly acidic pH and are mostly unstable at neutral pH. When cathepsins are outside the lysosomes or extracellularly they can be relatively rapidly and irreversibly inactivated. One exception is cathepsin S, which is still stable at neutral or slightly alkaline pH, thus retaining most of its activity (reviewed in Turk et al., 2012). In the acidic lysosomal milieu, cathepsin C is primarily an amino dipeptidase, cleaving two-residue units from the N-terminus of a polypeptide chain. Cathepsin C is also stable at higher pH and can act as a transferase and catalyze the reverse reaction (reviewed in Turk et al., 2001). The common feature of both cathepsins, proposed here as relevant target proteases of internalized tick cystatin OmC2, is that they are also stable and active at higher pH compared to other lysosomal cathepsins. This may indicate that internalized cystatin OmC2 preferentially bound to cathepsins S and C in vesicles which were not highly acidic but they contained high amount of these two proteases in their active forms.

Cathepsin S is a key protease involved in the processing of invariant chain, a MHC II-associated chaperone, bound to the peptide binding groove instead of antigenic peptides. When cathepsin S is inhibited, invariant chain could not be efficiently processed to CLIP, removed from MHC II and displaced by degraded antigen. By controlling the pace of Ii degradation, cathepsin S is able to influence MHC class II-mediated presentation of antigens to CD4^+^ T cells (reviewed in Blum et al., 2013; Roche and Furuta, 2015). The effect of cystatin OmC2 on DC-mediated proliferation of CD4^+^ T cells have already been published (Salát et al., 2010), at least in a mouse system, with the same tick inhibitor as we used here. Enriched mouse CD11c^+^ cells were pre-treated with cystatin OmC2 for 4 h and then co-cultured with CD4^+^ T cells from transgenic OT-II mice together with OVA. As shown by Salát and coworkers, cystatin OmC2 reduced DC-mediated proliferation of CD4^+^ T lymphocytes from transgenic mice that specifically recognize ovalbumin peptide 323–339.

On the other hand, we did not observe that internalized cystatin OmC2 bound to AEP, another lysosomal cysteine protease, although AEP is present in immature DC in which it participates in the MHC II-associated antigen processing and presentation (Sepulveda et al., 2009). Tick cystatins lack the AEP binding site, the SND motif, in their protein sequence. This sequence is present, for example, in human cystatin C (Alvarez-Fernandez et al., 1999) and in the cystatin Bm-CPI-2 from the nematode parasite *B. malayi* (Murray et al., 2005). We propose that the internalized tick cystatin OmC2, when present in addition to endogenous cystatin C, changes the proteolytic degradation and effective processing of antigens. Among others, cathepsin S is inhibited by cystatin OmC2 in late endosomes, i.e., where antigen processing and presentation is taking place. A crucial step in invariant chain processing, the formation of the αβ-CLIP complex (Driessen et al., 1999; Lindner, 2017), may be interrupted.

As for the inhibition of cathepsin C with cystatin OmC2 we hypothesize that the inhibition of dipeptidyl peptidase activity (cathepsin C sequentially removes dipeptides from the N-termini of protein and peptide substrates) could affect the processing of several other proteins (among them also serine proteases). Interestingly, cathepsin C is highly expressed in monocyte-derived DC (Hashimoto et al., 1999; Le Naour et al., 2001), but its specific function in these professional APC has not yet been established (Hamilton et al., 2008). We have confirmed a high content of cathepsin C in MUTZ 3-derived immature DC, although there were no differences in the fluorescence signal of labeled cathepsin C between immature DC and the cells activated with LPS (Supplementary Figure 1C). However, it has previously been reported that the activity of cathepsin C is increased in immature human DC but decreases rapidly as the DC mature (Ishri et al., 2004). Further, a reduced activity of cathepsin C was associated with the stimulation of the maturation of monocyte-derived DC by trimeric CD40L, TNF-α or *Streptococcus pyogenes* (Ishri et al., 2004). Conceivably, the cathepsin C expression is also related (Hamilton et al., 2008) to the demonstration that activated DC have a tumoricidal activity and express perforin and granzyme B (Stary et al., 2007).

In DC that are affected by the components of tick saliva, the diminished exoprotease activity of cathepsin C by cystatin OmC2 would lead not only to the inhibition of one particular cathepsin but would affect all other proteases in proteolytic cascades dependent on the proteolytic cleavage and activation by cathepsin C. Novel, non-cytotoxic role of a serine protease granzyme B, associated to the promotion of antigen uptake and the suppression of premature T cell activation, has been proposed in plasmacytoid DC (Jahrsdörfer et al., 2014; Fabricius et al., 2016).

Neutrophils, similarly as plasmacytoid DC (Gregorio et al., 2010) and NK cells (Carrega and Ferlazzo, 2012; Sojka et al., 2014), are not frequently observed in normal skin, but they are recruited in high numbers after skin injury (Wilgus et al., 2013). We further speculate that the internalization of cystatin OmC2 could take place in neutrophils and in NK cells when they are present in the dermis. A putative interaction of cystatin OmC2 with cathepsin C as a processing enzyme of serine proteases in the secretory granules of neutrophils or NK cells may affect their killing capacity. Besides, the secretion of active cathepsin C from neutrophils has been reported recently by Hamon et al. (2016). Hypothetically, salivary tick cystatin OmC2 could bind to the secreted active cathepsin C outside the immune cells, if both were present in the same tissue.

Cystatin OmC2 diminishes the antigen-specific proliferation of mouse CD4+ T cells (Salát et al., 2010). As reported, cystatin OmC2 reduced the production of TNF-α by 20% and IL-12 by 25% following the LPS-induced maturation of pretreated DC (Salát et al., 2010). Similarly, in mouse DC activated by LPS *in vitro*, sialostatin L interfered with the TLR-mediated release of IL-12 and TNF-α, but not IL-10, and impaired the antigen-specific CD4^+^ T cell proliferation (Sá-Nunes et al., 2009). Studies of the bone marrow-derived DC from cathepsin S^−/−^ mice further confirmed that the immunomodulatory actions of sialostatin L are mediated by the inhibition of cathepsin S (Sá-Nunes et al., 2009). In addition to the previously reported reduction in the secretion of IL-12 (Salát et al., 2010), we report here that the surface expression of the costimulatory molecule CD86 was diminished when immature DC were stimulated with LPS in the presence of cystatin OmC2. We can't explain how the internalized cystatin OmC2, acting on cysteine cathepsins inside the endocytic pathway of treated cells, might be directly connected to the changes in the surface expression of HLA-DR and CD86 molecules, observed in LPS-stimulated cells, but not in non-stimulated cells (in the absence of LPS). We assume that the maturation of cells was in a way changed by the presence of cystatin OmC2. Currently, the mechanism behind the observed effect of cystatin OmC2 cannot be explained, and whether the reduction of the surface expression of CD86 and the secretion of IL-12 is linked to the reduced proteolytic activity of one or more cysteine proteases remains to be elucidated. Immature but not mature DC secrete large amount of cystatin C (Zavašnik-Bergant et al., 2005). It is tempting to speculate that endogenous cystatin C could affect the DC from which it was secreted, thus causing a feedback effect on their maturation and the expression of costimulatory molecules such as CD86. However, our existing data do not support the putative autocrine effect of cystatin C.

Another type 2 cystatin, which is present in chicken egg white, induced TLR/MyD88 signaling when combined with IFN-γ. This resulted in the activation of the IκB kinase complex (IKK) and NF-κB pathway in macrophages that were infected with parasitic *Leishmania donovani* promastigotes (Kar et al., 2009, 2011). In our DC model, fluorescently labeled cystatin OmC2 was not retained on the cell surface, which would have indicated its possible binding as a ligand to one of the proteins on plasma membrane. Instead, it was efficiently internalized to late endosomes and lysosomes. At this point, it is only possible to speculate as to whether cystatin OmC2 exhibits intrinsic structural features that would enable it to be engaged in membrane receptor binding.

In summary, exogenous cystatin OmC2 entered the late endocytic compartments and acted effectively on cysteine proteases inside the MUTZ-3-derived immature human DC. Among these proteases, two cathepsins, S and C, which are involved in antigen processing and proteolytic cascades, were accessed. In addition, the expression of CD86, a costimulatory molecule that is associated with the maturation of DC, was diminished. We propose that the diminution of the activity of two key cysteine proteases contributes to the overall ability of tick saliva to affect the immune response of tick's host.

## Author contributions

Conceived the study, designed the experiments and wrote the paper: TZ. Performed the experiments: TZ, RV, and AS. Analyzed the data: TZ, RV, and MF. Contributed reagents/materials/analysis tools: TZ, RV, MF, JS, LG, and PK. Contributed insightful suggestions on the study: PK and BT. Supervised the project: TZ and BT.

### Conflict of interest statement

The authors declare that the research was conducted in the absence of any commercial or financial relationships that could be construed as a potential conflict of interest.
