# Supplementary material for: Salivary Tick Cystatin OmC2 Targets Lysosomal Cathepsins S and C in Human Dendritic Cells
Source: Front Cell Infect Microbiol. 2017 Jun 30;7:288. doi: 10.3389/fcimb.2017.00288 (PMC5492865; doi:10.3389/fcimb.2017.00288)
Supplement: Supplementary file 1 [file Table1.PDF]

SUPPLEMENTARY TABLE 1 | Viability of cells cultured in the presence of cystatin OmC2 for 1 or 3 h. Control: non-treated cells.

	**non-treated cells**	**cystatin OmC2**		
		**2 μM**	**12 μM**	**15 μM**
**0 h**	**95 ± 3**	–	–	–
**1 h**	**95 ± 3**	**93 ± 2**	**90 ± 3**	**90 ± 4**
**3 h**	**95 ± 2**	**90 ± 3**	**91 ± 4**	**88 ± 6**
