# Supplementary material for: Salivary Tick Cystatin OmC2 Targets Lysosomal Cathepsins S and C in Human Dendritic Cells
Source: Front Cell Infect Microbiol. 2017 Jun 30;7:288. doi: 10.3389/fcimb.2017.00288 (PMC5492865; doi:10.3389/fcimb.2017.00288)
Supplement: Supplementary file 2 [file Table2.PDF]

SUPPLEMENTARY TABLE 2 | Identification of (A) cathepsin S and cystatin OmC2, and (B) cathepsin C and cystatin OmC2, in selected excised bands from the IEF gel by mass spectrometry (peptide mass fingerprint). The identified peptides in the amino acid sequences of cathepsin S (A), cathepsin C (B) and cystatin OmC2 (A, B) are indicated.

A

Amino acid sequence of human cathepsin S

MKRLVCVLLVCSSAVAQLHKDPTLDHHWHLWKKTYGKQYKEKNEEAVRRLIWEK
NLKFVMLHNLEHSMGMHSYDLGMNHLGDMTSEEVMSLMSSLRVPSQWQRNITYKS
NPNRILPDSVDWREKGCVTEVKYQGSCGACWAFSAVGALEAQLKLKTGKLVSLSAQ
NLVDCSTEKYGNKGCNGGFMTTAFQYIIDNKGIDSDASYPYKAMDQKCQYDSKYRA
ATCSKYTELPYGREDVLKEAVANKGPVSVGVDARHPSFFLYRSGVYYEPSCTQNVN
HGVLVVGYGDLNGKEYWLVKNSWGHNFGEEGYIRMARNKGNHCGIASFPSYPEI

Measured m/z	Calculated MH+	Intensity	Dev. (Da)	Dev. (ppm)	Range	Sequence
956.516	956.516	4431.276	0	0.458	246 - 255	GPVSVGVDAR
998.499	998.494	2971.989	0.005	5.306	227 - 234	YTELPYGR
1066.558	1066.547	19189.411	0.011	10.065	256 - 263	HPSFFLYR
1100.573	1100.573	633.811	-0.001	-0.687	114 - 122	ILPDSVDWR
1665.746	1665.74	593.467	0.006	3.425	298 - 311	NSWGHNFGEEGYIR

Amino acid sequence of recombinant tick cystatin OmC2

TSIPGGWTRQDPTEARFLELAHFATSSQTEGREFYDTVVTVKEVETQVVAGMNYKLT
IEISPSVCKIGEVQYSAEQCVPKDAQQKSTCVAVIYHVPWQNQKSVTSYRCEHHHHH
H

Measured m/z	Calculated MH+	Intensity	Dev. (Da)	Dev. (ppm)	Range	Sequence
1771.851	1771.872	161.848	-0.021	-11.698	1 – 16	TSIPGGWTRQDPTEAR
1793.887	1793.882	3985.992	0.005	2.723	17 - 32	FLELAHFATSSQTEGR

B

Amino acid sequence of human cathepsin C

MGAGPSLLLAALLLLLSGDGAVRCDTPANCTYLDLLGTWVFQVGSSGSQRDVNCSV
MPQEKKVVVYLQKLDTAYDDLGNSGHFTIIYNQGFEIVLNDYKWFAFFKYKEEGSK
VTTYCNETMTGWVHDVLGRNWACFTGKKVGTASENVYVNIAHLKNSQEKYSNRLY
KYDHNFVKAINAIQKSWTATTYMEYETLTLGDMIRRSGGHSRKIPRPKPAPLTAEIQQ
KILHLPTSWDWRNVHGINFVSPVRNQASCGSCYSFASMGMLEARIRILTNNSQTPILSP
QEVVSCSQYAQGCEGGFPYLIAGKYAQDFGLVEEACFPYTGTDSPCKMKEDCFRYYS
SEYHYVGGFYGGCNEALMKLELVHHGPMAVAFEVYDDFLHYKKGIYHHTGLRDPF
NPFELTNHAVLLVGYGTDSASGMDYWIVKNSWGTGWGENGYFRIRRGTDECAIESI
AVAATPIPKL

Measured m/z	Calculated MH+	Intensity	Dev. (Da)	Dev. (ppm)	Range	Sequence
845.441	845.434	393.112	0.007	7.757	100 - 105	WFAFFK
1338.755	1338.728	723.728	0.027	20.074	238 - 249	NVHGINFVSPVR

Amino acid sequence of recombinant tick cystatin OmC2

TSIPGGWTRQDPTEARFLELAHFATSSQTEGREFYDTVVTVKEVETQVVAGMNYKLT
IEISPSVCKIGEVQYSAEQCVPKDAQQKSTCVAVIYHVPWQNQKSVTSYRCEHHHHH
H

Measured m/z	Calculated MH+	Intensity	Dev. (Da)	Dev. (ppm)	Range	Sequence
712.37	712.362	1904.649	0.007	10.449	102 - 107	SVTSYR
1200.634	1200.615	856.488	0.02	16.399	33 - 42	EFYDTVVTVK
1246.693	1246.671	654.42	0.021	17.255	56 - 66	LTIEISPSVCK
1467.749	1467.715	762.408	0.034	23.186	43 - 55	EVETQVVAGMNYK
1607.809	1607.773	1510.865	0.035	21.865	67 - 80	IGEVQYSAEQCVPK
1793.909	1793.882	18243.976	0.028	15.438	17 - 32	FLELAHFATSSQTEGR
1930.008	1929.964	1904.582	0.044	22.926	86 - 101	STCVAVIYHVPWQNQK
