# Supplementary material for: Salivary Tick Cystatin OmC2 Targets Lysosomal Cathepsins S and C in Human Dendritic Cells
Source: Front Cell Infect Microbiol. 2017 Jun 30;7:288. doi: 10.3389/fcimb.2017.00288 (PMC5492865; doi:10.3389/fcimb.2017.00288)
Supplement: Supplementary file 4 [file Image1.PDF]

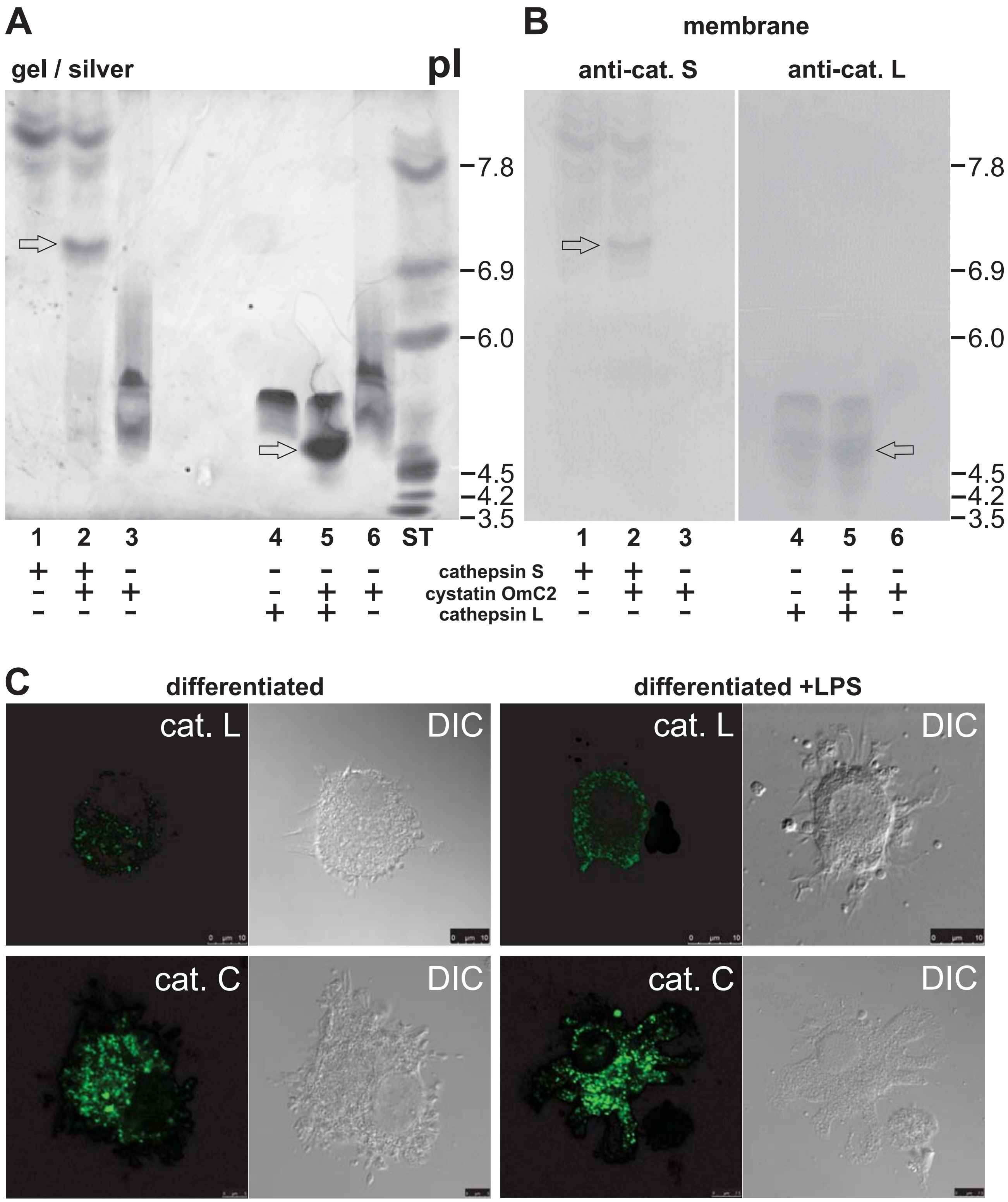

SUPPLEMENTARY FIGURE 1 | IEF (pH 3–10) of cathepsin S, cathepsin L and cystatin OmC2, expressed in *E. coli*, and the localization of cathepsins L and C in two populations of MUTZ-3 cells. (A) The proteins were stained with silver or (B) blotted to PVDF membranes and immunolabelled with anti-cathepsin S or anti-cathepsin L antibodies. The cystatin OmC2/cathepsin S complex and cystatin OmC2/cathepsin L complex that were formed are denoted (arrows). ST – standards. (C) Immunolabelled cathepsins L and C in fixed MUTZ-3 cells. Bars: 10 µm (cathepsin L), and 5 µm and 7.5 µm (cathepsin C).
