# Supplementary material for: Salivary Tick Cystatin OmC2 Targets Lysosomal Cathepsins S and C in Human Dendritic Cells
Source: Front Cell Infect Microbiol. 2017 Jun 30;7:288. doi: 10.3389/fcimb.2017.00288 (PMC5492865; doi:10.3389/fcimb.2017.00288)
Supplement: Supplementary file 5 [file Image2.PDF]

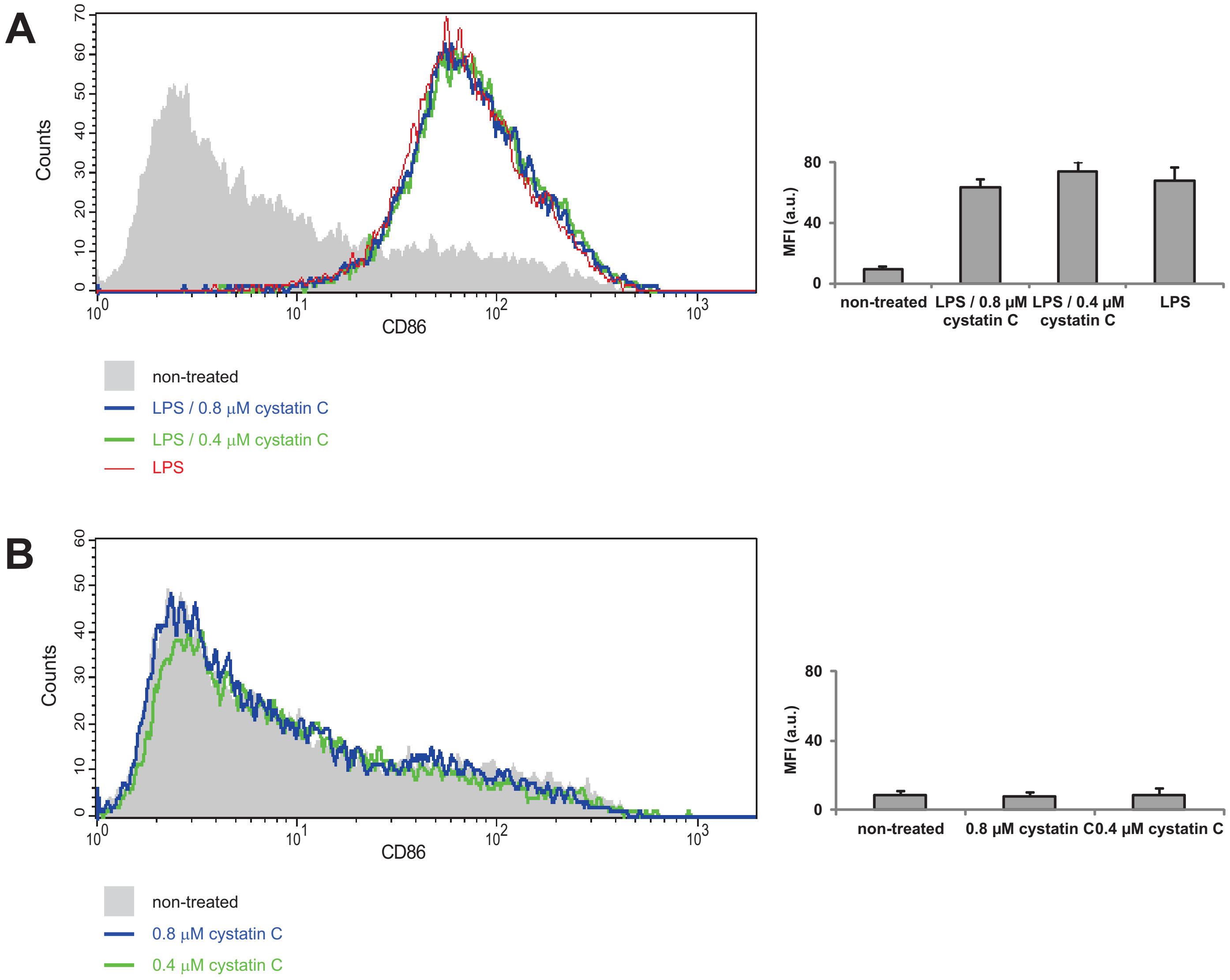

SUPPLEMENTARY FIGURE 2 | Effect of cystatin C on the expression of CD86. (A) LPS-induced maturation in the presence of cystatin C (*blue and green histograms*) is compared to non-treated cells (without LPS and cystatin C), and to LPS-induced maturation without cystatin C (*red histogram*). (B) The histograms show CD86 in cells that were cultured in the presence of cystatin C, but were not matured with LPS. Shadowed histograms represent the cells that weren't treated with LPS or cystatin C. A representative analysis of three independent biological replicates is shown. Mean fluorescence intensities (MFI) of labelled cell populations (geometric means) are shown in bar graphs.
