# Supplementary material for: Salivary Tick Cystatin OmC2 Targets Lysosomal Cathepsins S and C in Human Dendritic Cells
Source: Front Cell Infect Microbiol. 2017 Jun 30;7:288. doi: 10.3389/fcimb.2017.00288 (PMC5492865; doi:10.3389/fcimb.2017.00288)
Supplement: Supplementary file 6 [file Image3.PDF]

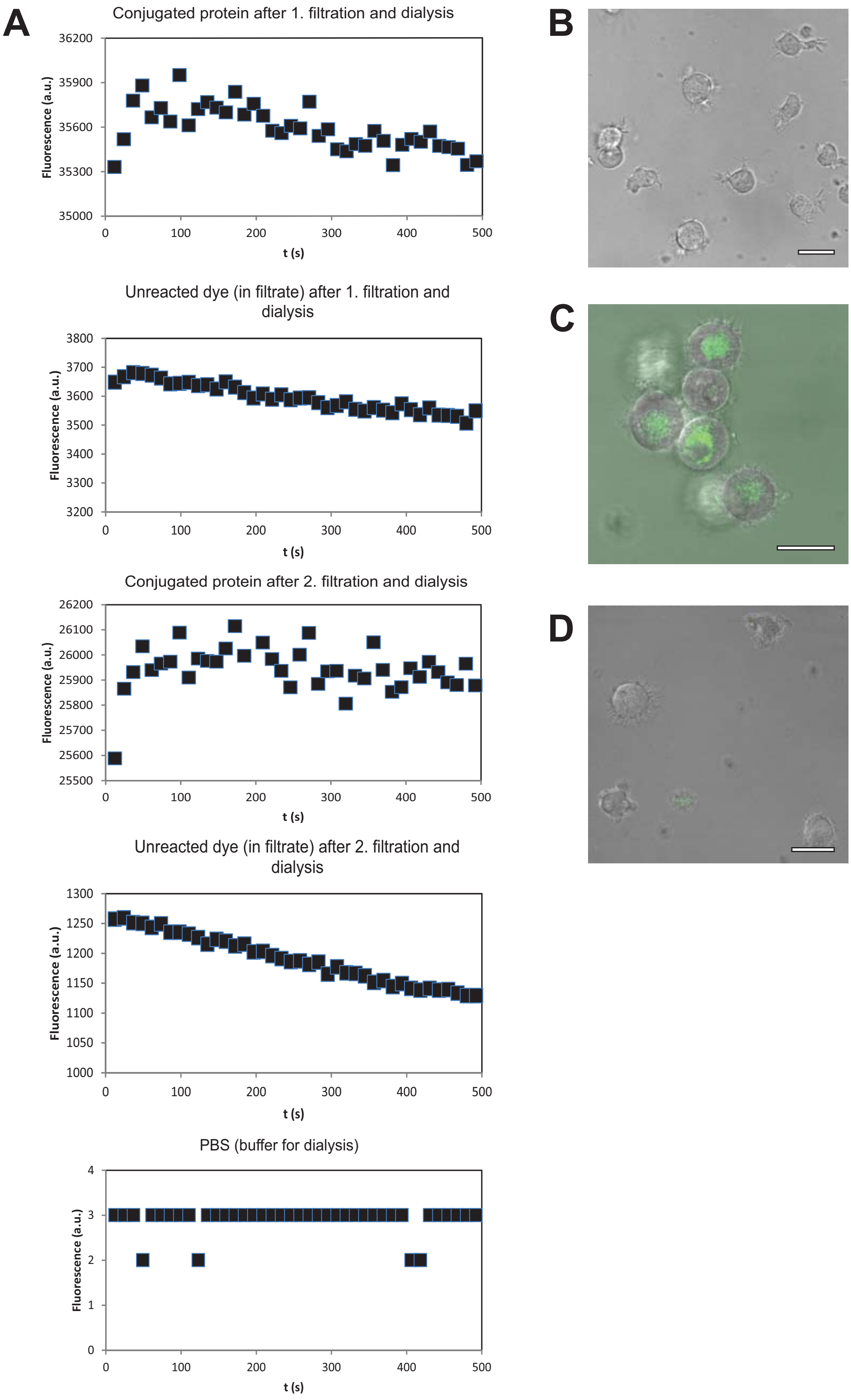

SUPPLEMENTARY FIGURE 3 | Isolation of fluorescently labelled cystatin OmC2 from unreacted Alexa Fluor 488 dye after gel filtration. The fluorescence was measured after two sequential membrane filtrations and dialyses (A). The fractions containing the conjugated cystatin OmC2 were compared to the fractions containing the unreacted dye and to PBS buffer before dialysis. The images of the non-fixed cells show: (B) non-treated differentiated MUTZ-3 cells, (C) cells cultured for 3 h in the presence of purified cystatin OmC2 conjugated to Alexa Fluor A488 (conjugated protein after 2. filtration and dialysis), and (D) cells cultured for 3 h with the same volume of filtrate containing the unreacted dye that remained after the last purification step (unreacted dye in filtrate after 2. filtration and dialysis). Bars: 20 µm.
