# Supplementary material for: Salivary Tick Cystatin OmC2 Targets Lysosomal Cathepsins S and C in Human Dendritic Cells
Source: Front Cell Infect Microbiol. 2017 Jun 30;7:288. doi: 10.3389/fcimb.2017.00288 (PMC5492865; doi:10.3389/fcimb.2017.00288)
Supplement: Supplementary file 7 [file Image4.PDF]

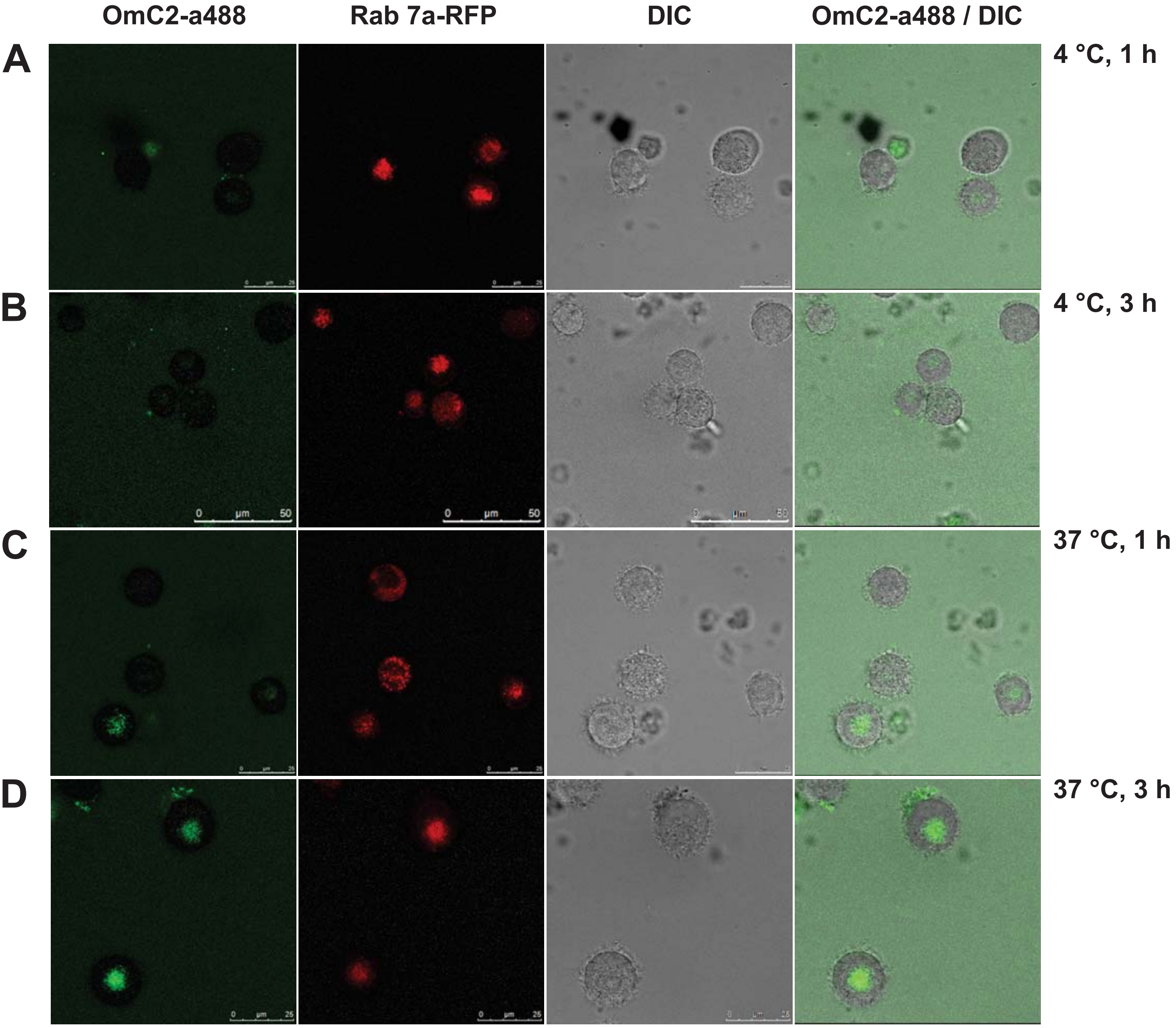

SUPPLEMENTARY FIGURE 4 | Localization of internalized cystatin OmC2 labelled with Alexa Fluor 488 (green fluorescence) in differentiated MUTZ-3 cells after 1 h (A, C) and 3 h (B, D) at 4 °C and 37 °C. Red fluorescence shows the localization of Rab 7a-positive vesicles in viable cells. Bars: 25 µm (A, C, D), 50 µm (B).
