# Supplementary material for: Salivary Tick Cystatin OmC2 Targets Lysosomal Cathepsins S and C in Human Dendritic Cells
Source: Front Cell Infect Microbiol. 2017 Jun 30;7:288. doi: 10.3389/fcimb.2017.00288 (PMC5492865; doi:10.3389/fcimb.2017.00288)
Supplement: Supplementary file 8 [file Image5.PDF]

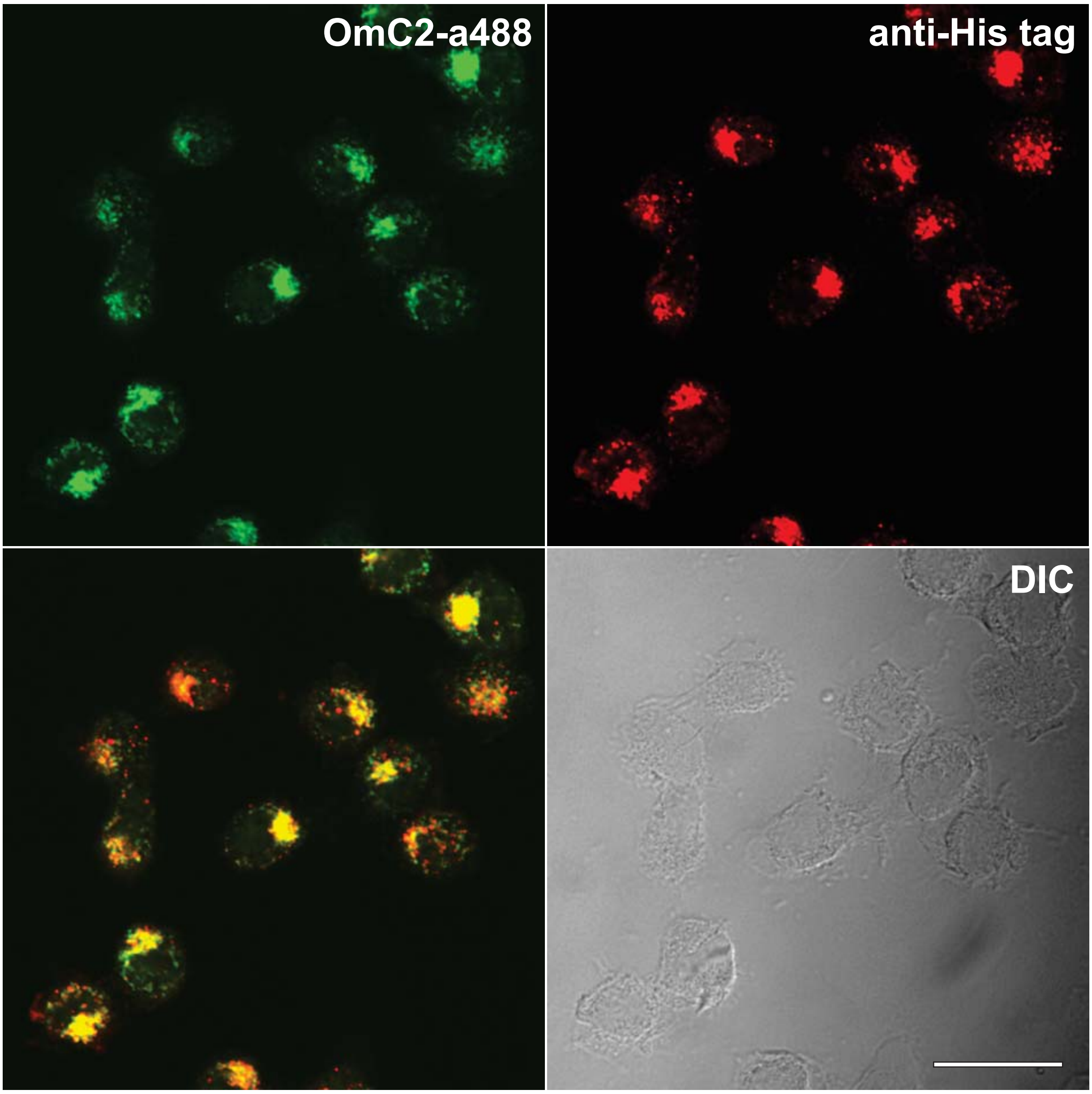

SUPPLEMENTARY FIGURE 5 | Localization of internalized cystatin OmC2 labelled with Alexa Fluor 488 (green fluorescence). Fixed differentiated MUTZ-3 cells were also labelled with anti-His tag antibody (red fluorescence). Bar: 25 µm.
